# ALS: An Organism-Wide Bioenergetic Failure Due to Mitochondrial Dysfunctions?

**DOI:** 10.3390/biom16081126

**Published:** 2026-08-01

**Authors:** Hiroshi Mitsumoto, Hélène Blasco, Philippe Corcia, Vincenzo Silani

**Affiliations:** 1 Department of Neurology, Neurological Institute of New York, Columbia University, 710 West 168 St., New York, NY 10032, USA; 2 Laboratoire de Biochimie et Biologie Moléculaire, Hôpital Bretonneau, CHU de Tours 2, Bd Tonnellé, 37044 Tours, France; helene.blasco@univ-tours.fr; 3 Université de Tours, INSERM, Imaging Brain & Neuropsychiatry iBraiN U1253, 37032 Tours, France; corcia@med.univ-tours.fr; 4 Centre de Reference Coordinateur SLA et Autres Maladies du Motoneurone, CHU Bretonneau 2 Boulevard TONNELLE, 37044 Tours, France; 5 Department of Neuroscience and Laboratory of Neuroscience, Istituto Auxologico Italiano, IRCCS, 20149 Milan, Italy; vincenzo@silani.com; 6 Department of Pathophysiology and Transplantation, “Dino Ferrari” Center, Università degli Studi di Milano, 20122 Milan, Italy

**Keywords:** ALS, amyotrophic lateral sclerosis, MND, motor neuron disease, hypothalamus, frontotemporal systemic disease, multisystem involvement, metabolic disease, mitochondria, mitochondrial associated membrane (MAM), bioenergetics

## Abstract

Amyotrophic lateral sclerosis (ALS) is a devastating and invariably fatal disease for which currently available disease-modifying therapies provide only modest benefit. Defining its underlying pathogenesis is therefore essential for the development of effective treatments. Increasing evidence indicates that ALS is not restricted to motor neurons but involves multiple neuronal and glial systems, extending to peripheral organs, often at subclinical levels. These multisystem alterations may precede overt neurological symptoms by years and are accompanied by metabolic disturbances, including progressive weight loss and hypermetabolism. In peripheral tissues, ongoing cellular turnover and associated immune and inflammatory responses may further increase energy demand. Within this framework, mitochondrial dysfunction emerges as a central mechanism underlying impaired bioenergetics and systemic metabolic failure. Mitochondria not only regulate energy production but also contribute to oxidative stress, which in turn exacerbates mitochondrial injury, creating a self-amplifying cycle. Importantly, many genetic forms of familial ALS directly affect mitochondrial pathways, and similar biochemical abnormalities are observed in sporadic ALS. These shared features suggest that mitochondrial dysfunction represents a common pathway across ALS subtypes. Targeting upstream mechanisms of mitochondrial impairment may therefore provide a unifying strategy for understanding ALS pathogenesis and developing effective therapies.

## 1. Introduction

Charcot described the prognosis of ALS most explicitly in his lectures: “The prognosis, up to the present, is of the gloomiest. There does not exist, so far as I am aware, a single example of a case where, the group of symptoms just described having existed, recovery followed. Is this doom final? The future alone can decide”, quoted in Mitsumoto et al. [1]. Charcot’s statement, made more than 150 years ago, may still hold true today, even if the use of anti-SOD1 antisense oligonucleotides (ASOs) has begun to alter this outlook, particularly in genetic forms of ALS. Furthermore, multidisciplinary ALS clinics have substantially improved patient and caregiver management, allowing us to provide considerably more comprehensive care for individuals with ALS.

ALS has a profound impact not only on individuals living with this devastating disease and their families, but also on the physicians who care for them. It remains one of the most challenging and intractable neurological disorders. A small number of disease-modifying medications are currently approved for ALS; however, their effects on disease progression are modest. Intensive clinical trials aimed at developing more effective therapies are ongoing worldwide. Nevertheless, elucidating the underlying causes and pathogenic mechanisms of ALS remains essential for the development of truly effective treatments.

Increasing evidence suggests that ALS is not solely a disorder of motor neuron degeneration, but rather a complex multisystem disease involving multiple neuronal networks with prominent central metabolic alterations, alongside systemic metabolic disturbances. In this review, we summarize evidence demonstrating widespread multisystem involvement of the central nervous system (CNS) in ALS, extending beyond the traditional concept of motor system degeneration [1]. We also review evidence that multiple peripheral organs are affected, often at subclinical levels. Emerging data suggest that metabolic derangements and widespread mitochondrial dysfunction represent fundamental components of ALS pathophysiology. Understanding these interactions may broaden our perspective on ALS mechanisms and provide new directions for future research.

## 2. Review Methods

The literature included in this review was primarily identified through searches of the PubMed/Medline database, and further Scopus and Web of Science Core Collection databases. The principal search terms were ALS amyotrophic lateral sclerosis, combined with additional keywords relevant to specific topics of interest. The search strategy was then progressively refined and expanded based on emerging results. References were further expanded through cross-referencing until comprehensive coverage of the literature was achieved. ALS cases were classified using the conventional categories of sporadic ALS (sALS) and familial ALS (fALS). ChatGPT (OpenAI; GPT-4 and GTP-5.5 software was used) was used in a limited capacity for language editing and clarity of expressions or definitions when needed.

## 3. Multisystem Neuronal Involvement

Motor neuron involvement in ALS has historically been viewed within a spectrum of subacute or chronic multiple system degeneration that shows a predilection for specific components of the motor system [1]. Widespread involvement of motor neurons, including anterior horn cells and brainstem motor neurons, has therefore long been recognized as a defining pathological feature of the disease. Over time, however, accumulating clinical, neuropathological, neurobiological, and genetic evidence has led to the recognition of an additional essential cellular participant in ALS, namely neuronal supportive cells. Traditionally, glial cells were thought to merely fill the space left after tissue loss or neuronal cell death. However, our understanding of glial cells—more specifically, astrocytes—has advanced enormously. Astrocytes are now recognized as essential for a wide range of functions, from neuronal metabolism and structural support to the regulation of synaptogenesis and synaptic transmission; moreover, they exhibit significant regional heterogeneity within the brain [2]. Microglia and astrocytes, through crosstalk with peripheral immune cells, can exert both neuroprotective and deleterious effects, resulting in a highly nuanced spectrum of neuronal and non-neuronal interactions [3]. Neuron–astrocyte metabolic interactions play a critical role in the selective motor neuron loss observed in ALS, particularly in glutamatergic motor neurons [4]. In this context, astrocytes contribute to increased extracellular glutamate levels in ALS, and conversely, are themselves susceptible to glutamate-mediated toxicity [5]. Furthermore, interactions between motor neurons and astrocytes are clearly impaired in ALS [6]. Most importantly, astrocytes exert non-cell-autonomous toxicity in ALS [7] and thus, induce neuronal cell death, as demonstrated in several in vitro studies [8,9,10]. Therefore, astrocytes appear to exert overarching control over motor neurons and other vulnerable neuronal populations while also being vulnerable to excitotoxic, metabolic, and oxidative stress [11].

For the neuronal side, recent advances have established the concept that ALS affects broader neuronal networks beyond the motor system. In particular, frontotemporal neuronal involvement has become firmly integrated into the ALS disease spectrum. The clinical and neuropathological entity of frontotemporal dementia was first described by Mitsuyama [12]. Subsequent work recognized a broader pathological continuum linking ALS and frontotemporal degeneration as manifestations of a multisystem neurodegenerative disorder [13]. A major breakthrough occurred with the discovery of common ubiquitinated TDP-43 inclusions in ALS and FTD [14], followed by the identification of the C9orf72 mutation as a cause of familial ALS, which further expanded the recognized CNS involvement to include frontotemporal cognitive impairment [15,16]. Frontotemporal cognitive impairment has been reported in sporadic ALS cohorts, with 6.5% of individuals scoring below the cutoff for frontotemporal lobar dementia, 54.2% within a range consistent with ALS with mild cognitive impairment, and 39.2% within the normal range based on the ALS Cognitive Behavioral Screen. Thus, frontotemporal cognitive impairment is now also widely accepted as part of sporadic ALS (sALS) [17].

More recently, hypothalamic involvement has emerged as another important component of ALS pathology, adding a new dimension to the concept of multisystem disease. In 2014, the Appel research group reported for the first time the presence of TDP-43 pathology—the hallmark of sALS—in the hypothalamus of some ALS cases [18]. This observation was subsequently confirmed by additional neuropathological studies [19,20]. Structural imaging studies soon followed, demonstrating hypothalamic volume loss on magnetic resonance imaging (MRI) [21,22,23], particularly involving atrophy of the anterior–superior hypothalamic subregion [24]. Functional MRI studies have also shown decreased connectivity involving the hypothalamus [25]. A sophisticated study measuring peripheral lipid metabolism, expressed as the angiopoietin-like protein (ANGPTL) system, correlated with hypothalamic atrophy, suggesting that peripheral lipid metabolism is linked to dysfunction of a specific hypothalamic circuit [26]. Another recent study indicated that the hypothalamus was an early site of mitochondrial failure and neuroimmunological disruption [27].

These findings indicate that ALS affects not only motor and frontotemporal cognitive–behavioral networks but also hypothalamic circuits responsible for regulating fundamental physiological processes, including metabolic, endocrine, immunological, and behavioral functions (see below regarding the relationship to weight loss). Why these particular neuronal systems are selectively vulnerable in ALS remains an important question. One possible explanation is that these neurons share exceptionally high energy demands while being particularly vulnerable to disturbances in cellular energy homeostasis [28]. Table 1 summarizes their neuronal characteristics and metabolic requirements compared with cortical interneurons and primary sensory neurons [29,30,31,32]. In addition, subtle involvement of the visual/retinal and olfactory systems has been reported, further supporting the concept of multisystem CNS involvement in ALS. These findings may represent additional extra-motor manifestations of the disease (see below).

## 4. ALS Is a Systemic Disease

Dr. Stanley Appel was among the first investigators to describe ALS as a systemic disease, based on the observations of extensive systemic pro-inflammatory responses in ALS [33]. In the following sections, we describe how multiple physiological systems are involved in ALS beyond the traditionally recognized motor neuronal degeneration.

### 4.1. Global and Sleep Issues

Based on a questionnaire study conducted among Scottish individuals with ALS or motor neuron disease (MND), patients reported numerous non-motor symptoms, including pain, fatigue, gastrointestinal problems, sleep disturbances, mood changes, anxiety, excessive or problematic saliva, apathy, emotional lability, cognitive complaints, and sexual dysfunction [34]. These symptoms suggest the presence of widespread systemic disturbances, many of which may be related to hypothalamic regulation.

Because a substantial portion of daily life is spent sleeping, sleep disturbances represent an important but often underrecognized issue in individuals with ALS. Despite frequently reporting “no sleep problems”, objective assessments have demonstrated that sleep is often significantly disturbed in individuals with ALS [35,36]. As the disease progresses, abnormal sleep behavior may increasingly reflect broader hypothalamic dysfunction [19]. In addition, excessive daytime sleepiness appears to occur more frequently in individuals with fALS than in those with sALS [37,38]. “Although sleep disturbances are a clinical issue in individuals with ALS, it is critically important to exclude underlying respiratory insufficiency and nocturnal hypercapnia, which may remain clinically underrecognized but can significantly impair sleep quality” [39].

### 4.2. Cutaneous System

Charcot noted that individuals with ALS, even when profoundly paralyzed and confined to bed, rarely developed decubitus ulcers, which were extremely common in chronically bedridden patients with other neurological or medical conditions at that time [1]. Subsequent investigators attempted to explain this puzzling observation and suggested that the relative absence of pressure ulcers might be related to preserved pain sensation and intact autonomic nervous system function.

Dr. S. Ono and colleagues [40] were the first to systematically investigate this phenomenon. Their group described structural differences in dermal collagen in ALS skin. Collagen bundles were reduced in amount and more loosely organized compared with the controls. Electron microscopic analysis revealed a significant negative correlation between disease duration and collagen fibril diameter in ALS patients. In addition, a marked increase in amorphous material was observed within the dermis. These collagen abnormalities were further characterized through biochemical analyses [41]. Similar collagen alterations were later confirmed by other independent investigators [42].

Subsequent studies demonstrated that ALS-related protein pathology is also present in the skin. TDP-43 and ubiquitin-positive inclusions, along with rare FUS inclusions, have been identified in skin biopsies, recapitulating the pathological hallmarks observed in the nervous system [43,44]. In efforts to identify potential mechanistic links between neuronal degeneration and cutaneous alterations, matrix metalloproteinase-9 (MMP-9) was found to be significantly elevated in both cerebrospinal fluid (CSF) and skin, suggesting a possible connection between neuronal and skin pathology [45]. TDP-43 inclusions have also been detected in skin biopsies well before the onset of clinical symptoms in presymptomatic carriers of familial ALS mutations [46]. In one striking case, an individual with positive TDP-43 inclusions in a skin biopsy developed clinically diagnosed ALS 26.5 years after the biopsy [46]. In other studies, mitochondrial abnormalities have also been identified in skin cells. Keratinocyte mitochondria in skin biopsies from five individuals with sporadic ALS demonstrated marked ultrastructural abnormalities, including reduced mitochondrial size, decreased area and perimeter, and disruption of cristae and outer membranes [47]. Similarly, fibroblasts cultured from skin biopsies of patients with sporadic ALS exhibit significant mitochondrial abnormalities (see Mitochondrial section below). Interestingly, an artificial three-dimensional (3D) skin model generated from patient-derived skin shows TDP-43 aggregates and proinflammatory, pyroptotic programmed cell death, suggesting a potential tool for future ALS research [48].

### 4.3. Hepatic System

Nakano and colleagues [49], neuropathologists with particular expertise in ALS, reported mitochondrial structural abnormalities in liver biopsies obtained from 21 individuals with ALS. The authors appear to have specifically investigated the possibility of extra-motor involvement. Their ultrastructural analyses revealed several striking abnormalities, including giant mitochondria, intramitochondrial paracrystalline inclusions, disorganization of the lamellar structure of the rough endoplasmic reticulum, and increased numbers of smooth endoplasmic reticulum. They also noted a relatively high incidence of mild liver dysfunction among these patients [49]. Subsequently, the potential presence of non-alcoholic fatty liver disease in ALS was explored. MRI-based analyses have reported findings consistent with non-alcoholic fatty liver disease or steatosis in approximately 77% of individuals with various forms of MND, including sALS and fALS [50,51].

### 4.4. Skeletal Muscle System

Skeletal muscle is the largest organ system in the body, accounting for approximately half of the total body mass. In ALS, skeletal muscle undergoes relentless and progressive denervation atrophy secondary to motor neuron degeneration. An important question, however, is whether skeletal muscle pathology in ALS reflects purely denervation atrophy or whether intrinsic muscle abnormalities also contribute to the disease process.

During submaximal aerobic exercise testing, skeletal muscle in individuals with ALS demonstrates a significantly increased oxygen cost (mL oxygen/kpm). In addition, several circulating metabolites—including plasma free fatty acids, β-hydroxybutyrate, and esterified carnitine—are abnormally elevated in individuals with ALS [52]. In fact, ketone body and free fatty acid levels are higher in individuals with ALS compared to controls at rest, particularly when BMI is higher [53]. Lactate measurements in ALS patients have shown elevated lactate levels both at rest and during incremental bicycle exercise testing, indicating increased anaerobic metabolism, with lactate production exceeding clearance. These findings further suggest impaired mitochondrial oxidative metabolism in ALS [54].

### 4.5. Hematologic System

Platelet mitochondria from individuals with ALS demonstrate ultrastructural abnormalities, including altered granules and vacuoles. These changes have been interpreted as reflecting modest mitochondrial dysfunction [55]. Further investigations by the same group demonstrated perturbations in mitochondrial membrane potential, mitochondrial depolarization, and increased apoptosis in ALS platelets, suggesting that platelet mitochondria are functionally impaired [56]. Furthermore, in a small number of individuals with ALS, platelet metabolomics and fatty acid analyses showed distinct alternation compared with the controls [57]. Circulating mononuclear cells from ALS patients also exhibit mitochondrial abnormalities. In these cells, complex IV (cytochrome c oxidase) activity is reduced despite increased mitochondrial content, likely reflecting a compensatory response [58]. Similarly, studies examining mitochondrial function in lymphocytes have reported complex I (NADH) dehydrogenase deficiency, reduced intracellular ATP levels, and compensatory increases in intracellular ADP content [59]. Furthermore, widespread metalloproteinase and their tissue inhibitor alterations were found by multiplex ELISA analysis, demonstrating diffuse enzymatic variations in mesenchymal stem cell compartments, suggesting that ALS is confirmed to be a systemic disease, not restricted to the nervous system, but also affecting the BM stromal compartment, even in sporadic cases [60].

### 4.6. Autonomic System

#### 4.6.1. General Features

A variety of autonomic symptoms have been reported in individuals with ALS at the time of diagnosis and tend to progress over the course of the disease. These observations suggest that autonomic dysfunction represents an intrinsic non-motor feature of ALS. Moreover, a greater autonomic symptom burden has been associated with poorer prognosis [61]. A questionnaire specifically designed to evaluate autonomic dysfunction revealed significantly elevated gastrointestinal and urinary symptom subscores in ALS patients [62]. Subclinical impairment of sympathetic sudomotor function has also been reported [62,63,64]. Parasympathetic dysfunction has also been documented. Cardiovagal responses were found to be impaired, and ultrasonographic studies demonstrated significant vagus nerve atrophy in ALS patients [63]. Individuals with bulbar-onset ALS appear to exhibit greater impairment of sympathetic sudomotor function [64]. Histological analyses of cutaneous and autonomic nerves have revealed the loss of intraepidermal nerve fibers and Meissner corpuscles, a reduced density of pilomotor nerves involving both cholinergic and noradrenergic fibers, and decreased vascular density in the dermis [65].

#### 4.6.2. Cardiovascular System

Cardiac muscle cells have among the highest oxygen demand, and like neurons, are post-mitotic; therefore, they are biologically vulnerable cell types. A recent study reported that the combined use of cardiac troponin T (cTnT) and neurofilament light (NfL) improves the discrimination of ALS from other neurodegenerative conditions, suggesting increased cardiac troponin release in ALS [66,67]. For autonomic testing, the most consistent findings from cardiovascular autonomic testing in ALS include reduced heart rate variability and sympathetic overactivity, often manifested as mild baseline tachycardia [68,69].

These observations have led to the interpretation that basic cardiovascular autonomic control may remain relatively intact in ALS, but with evidence of sympathetic predominance and vagal withdrawal accompanied by reduced baroreflex sensitivity. Interestingly, similar physiological patterns are observed in essential hypertension [70]. A large multicenter study conducted in Italy further demonstrated that hypertension, heart disease, and hematological disorders were independently associated with shorter survival in ALS [71]. These findings warrant further investigation into the role of cardiovascular comorbidities in ALS prognosis.

#### 4.6.3. Gastrointestinal System

Gastrointestinal autonomic function has been evaluated through measurements of gastric and colonic transit. One study reported significantly delayed gastric emptying in 15 out of 18 ALS patients, with an average emptying time of 218 ± 48 min compared with 138 ± 34 min in healthy controls, and similarly delayed colonic transit times were also noted [72]. Upper esophageal sphincter (UES) pressure was higher in all 13 ALS patients studied [73]. Constipation is therefore common in ALS. In a study of 66 patients, constipation increased from 33% at the baseline to 65% following diagnosis [74]. Because constipation in ALS is multifactorial, it is often difficult to identify a single cause; however, gastrointestinal autonomic dysfunction likely contributes to this symptom.

### 4.7. Exocrine System (Pancreatic, Salivary, and Sweat Glands)

It has been shown that exocrine gland function was impaired in patients with ALS. A later study confirmed that salivary gland function, including both parotid and submandibular secretion, was significantly reduced in ALS, although often at subclinical levels [75]. Abnormal sweat gland function has also been demonstrated using sympathetic sudomotor testing (see above).

### 4.8. Endocrine and Hormonal System

Complex hormonal and metabolic regulation is primarily coordinated by the hypothalamus, although extra-hypothalamic brain regions also participate in these processes. Many of these regions contain receptors for metabolic hormones, suggesting that neuroendocrine signaling pathways may contribute to biological processes underlying the pathogenesis of ALS and frontotemporal dementia (FTD) [76]. In addition, the sexually dimorphic nature of ALS suggests that endocrine, hormonal, or neuroglial factors may influence disease susceptibility or progression [77,78]. It is well-known that after menopause, the male predominance decreases, and bulbar-onset ALS becomes more frequent in older men than in younger men. Despite these considerations, there is currently no clear evidence of systemic endocrine or hormonal involvement in ALS.

### 4.9. Immune System and Inflammatory Responses

Immune function is regulated by a distributed neural control network centered on the hypothalamus and brainstem, with efferent regulation mediated primarily through the autonomic nervous system and neuroendocrine pathways. Neurodegeneration in ALS is accompanied by a well-characterized neuroinflammatory response within the central nervous system. At the same time, peripheral inflammatory markers are activated during the course of the disease [79]. Evidence of systemic immune activation has also been reported in individuals with ALS [80]. Increased production of CD8^+^ T cells and natural killer T (NKT) cells has been interpreted as suggesting an immune response to unidentified endogenous proteins or possibly viral antigens. A population-based study conducted in Italy examining peripheral immune markers demonstrated dysregulation of systemic immunity and elevated innate immune responses, which were associated with faster disease progression and reduced survival [81]. More recent work has further suggested interactions between central and peripheral immune systems in ALS. Kim et al. [82] reported the activation of macrophages and clonally expanded CD8^+^ T cells, suggesting bidirectional communication between peripheral immune cells and central nervous system inflammatory pathways. In addition, activated microglia may promote the recruitment of peripheral inflammatory cells into the CNS in ALS [83]. Whether ALS has an autoimmune component remains uncertain [84]. The immune system is highly complex. For example, interleukin-2 (IL-2) is a cytokine that regulates immune responses and has been used in cancer treatment. Recently, low-dose IL-2 was tested in individuals with ALS for potential disease-modifying effects and showed possible benefits in unadjusted analyses, suggesting that the modulation of immune pathways may have therapeutic potential [85].

#### Inflammatory Markers

Several circulating inflammatory markers are elevated in ALS. Levels of C-reactive protein (CRP), fibrinogen, erythrocyte sedimentation rate (ESR), and the neutrophil-to-lymphocyte ratio (NLR) have all been reported to be significantly increased in individuals with ALS compared with controls [86]. The elevation of CRP has been confirmed by subsequent studies [87,88]. Other investigators have also reported that NLR may serve as a useful marker for detecting systemic inflammatory responses in ALS [89]. These acute-phase reactants appear to correlate with disease burden, rates of progression, and survival, supporting the concept that ALS is associated with widespread systemic pro-inflammatory responses [90]. Interestingly, similar patterns of inflammatory marker elevation have not been consistently observed in other neurodegenerative diseases such as Alzheimer’s disease, Parkinson’s disease, or frontotemporal dementia, suggesting that this inflammatory signature may be relatively specific to ALS. It remains unclear whether these immune responses represent secondary reactions to ongoing neurodegeneration or reflect a primary immunological process in ALS. Obviously, further studies are needed.

### 4.10. Visual and Retinal System

The visual and retinal system represents a direct extension of the CNS. Using high-resolution spectral-domain optical coherence tomography (OCT), Ringelstein and colleagues [91] reported a distinctive pattern of retinal alterations in ALS, including subtle reductions in macular thickness and the retinal nerve fiber layer (RNFL), as well as marked thinning of the inner nuclear layer. These findings suggest that neurodegeneration in ALS extends beyond the motor system. In the same year, an individual with C9orf72-associated ALS was reported to have abnormalities in the anterior visual pathway, characterized by involvement of cone bipolar cells within the inner nuclear layer of the retina. These pathological changes were thought to explain subtle visual functional deficits observed in this individual [92]. Subsequent studies have confirmed retinal structural abnormalities in ALS, including alterations in retinal layers and increased thickness of the choroidal layer [93,94,95].

An intriguing recent study using data from approximately 54,000 participants in the UK Biobank who underwent OCT examination and were followed for approximately 14 years reported that 72 individuals eventually developed ALS. Retinal abnormalities detected by OCT were observed during the preclinical stage in some individuals, suggesting that retinal OCT imaging may potentially identify early neurodegenerative changes prior to clinical disease onset [96]. Another important report described a unique opportunity to directly examine retinal spheroids and axonal pathology in individuals with ALS. These findings appear to replicate neuronal pathology considered characteristic of ALS in the CNS [97].

### 4.11. Olfactory System

The olfactory system also represents a direct extension of the central nervous system. Olfactory dysfunction has long been recognized in individuals with Guamanian ALS–Parkinsonism–dementia [98] and other neurodegenerative disorders [99]. More recently, significant olfactory dysfunction has also been documented in individuals with ALS [100], particularly in association with cognitive and behavioral impairment [101,102]. Neuroimaging studies have provided additional insight into the structural correlates of olfactory dysfunction in ALS. One study demonstrated that olfactory impairment in ALS was significantly associated with atrophic changes in the left orbitofrontal cortex, including the gyrus rectus and medial orbital gyrus, as well as in the right hippocampus on voxel-based MRI analysis [103].

### 4.12. Bone System

A comprehensive bone health study evaluating bone mineral density and other metabolic parameters demonstrated that individuals with ALS, particularly females, exhibit significantly poorer bone health compared with healthy controls [104]. However, relatively few studies have examined bone metabolism and skeletal health in ALS.

### 4.13. Summary of Multisystem Involvement

Collectively, these observations across multiple organ systems indicate that ALS is not confined to motor neurons but represents a multisystem disorder, as summarized in Table 2. Structural pathology in peripheral tissues, mitochondrial dysfunction in circulating cells and other tissues, and dysregulation of autonomic, endocrine, immune, and sensory systems together suggest that widespread disturbances of cellular energy homeostasis and metabolism may play a central role in ALS pathogenesis.

## 5. ALS Is a Metabolic Disease

Metabolic diseases are pathological conditions that arise from defects in biochemical pathways regulating the synthesis, breakdown, transport, or storage of metabolites required for cellular and organismal homeostasis. Increasing evidence suggests that disturbances in systemic energy metabolism represent a central feature of ALS pathophysiology. In this section, we begin with the severe weight loss in individuals with ALS.

### 5.1. Body Weight Loss

Severe weight loss in individuals with ALS was noted by Dr. Forbes Norris in 1979 [105], who is regarded as one of the founding figures in ALS research in the United States; he used the term ALS cachexia to emphasize its severity. Body weight reflects an integrated metabolic state shaped by multiple interacting determinants, including genetic, socioeconomic, environmental, medical, psychological, and lifestyle factors. When analyzing the effects of body weight, these factors must be considered for proper interpretation.

Stambler and colleagues [106] were the first to report that greater weight loss during the two months prior to study entry into a ciliary neurotrophic factor (CNTF) clinical trial predicted shorter survival. Although this observation may have been overshadowed by concurrent changes in %FVC and serum chloride, it represents one of the earliest identifications of a relationship between weight loss and survival in ALS. Subsequent studies have consistently confirmed this association between weight loss and reduced survival [107]. Analysis of the large pooled PRO-ACT database, which integrates data from multiple clinical trials, also demonstrated shorter survival associated with weight loss, although this effect was observed primarily in men. In that study, impaired respiratory function and weight loss were major contributors to survival differences, underscoring the importance of considering sex-specific factors in ALS [108]. A population-based study further showed that approximately two-thirds of individuals with ALS had already experienced weight loss at the time of diagnosis. Notably, this occurred independently of dysphagia and was associated with shortened survival [109]. The authors suggested that weight loss in ALS is likely multifactorial and may vary substantially among individuals. Nearly half of the 93 individuals with ALS in another study were found to be malnourished, which was independently associated with poor survival [110]. Loss of appetite has also been reported to be highly prevalent and is significantly associated with weight loss and reductions in fat mass [111]. A study from a nationwide cohort of approximately 24,000 individuals with ALS in China showed that 31.7% were diagnosed with dysphagia, and multivariate analyses indicated that malnutrition and weight loss were significantly associated with disease status [112]. Lactate is increasingly recognized as an energy substrate in ALS. A recent study showed that lower blood lactate is associated with increased risk of earlier death and greater weight loss in ALS, suggesting that lactate is a prognostic biomarker of nutritional status [113].

More detailed evaluations of body compositions, such as measurements of body mass, fat mass, and fat-free mass, has provided additional insight into how individuals with ALS lose body mass over the course of the disease [114]. Several physiological measurements have been used to assess energy balance in ALS, including bioelectrical impedance analysis (BIA) [115], total daily energy expenditure (TDEE) and resting energy expenditure (REE) [116,117]. These approaches provide important information regarding how individuals with ALS consume and replenish energy. Management of weight loss is therefore of paramount importance in ALS. A detailed discussion of therapeutic strategies will be addressed elsewhere; however, it is noteworthy that riluzole has been reported to attenuate weight loss in both early and late stages of the disease [118], and this important observation warrants further investigation.

#### 5.1.1. Preclinical Influence of Weight Loss

Evidence suggests that alterations in body weight may precede the clinical onset of ALS. A large prospective cohort study involving more than half a million participants of each sex found that lower baseline body mass index (BMI), measured years before diagnosis, was associated with a higher risk of ALS. For every 5-unit increase in BMI, ALS incidence was approximately 21% lower. Compared with individuals with a healthy BMI, ALS incidence was significantly lower among overweight and obese individuals [119]. A population-based case–control study from the ALS Registry Swabia in Germany also demonstrated a marked decrease in BMI beginning approximately 10 years before disease onset among ALS cases. Furthermore, weight loss was strongly associated with shorter survival in ALS patients [120]. These findings suggest that alterations in body weight may occur many years before the clinical manifestation of ALS. Similarly, another investigation showed that ALS participants experienced progressive weight loss during the five years preceding study enrollment. Individuals whose BMI trajectory demonstrated substantial weight loss in the decade before study entry had the poorest survival, supporting an association between lower preclinical BMI and ALS risk and prognosis [121]. Further prospective studies are needed to better characterize the temporal relationship between weight loss and ALS development.

#### 5.1.2. Relationship Between Weight Loss and Hypothalamic Involvement

An important study examined the relationship between hypothalamic atrophy and weight loss in individuals with sALS and fALS and presymptomatic mutation carriers. The investigators found marked hypothalamic atrophy in both sALS and symptomatic mutation carriers, particularly affecting the anterior and posterior hypothalamic regions [122]. Hypothalamic volume was significantly correlated with BMI in individuals with sALS and even more strongly in individuals with fALS. Notably, hypothalamic atrophy associated with BMI was also present in presymptomatic mutation carriers [122]. Another study reported a significant association between hypothalamic atrophy and shortened survival, although correlations with other clinical measures were more limited, likely due to the relatively small sample size [21].

#### 5.1.3. Non-Hypothalamic Causes of Weight Loss

Weight loss in ALS may also arise from mechanisms independent of hypothalamic dysfunction. A meta-analysis of ALS genome-wide association studies (GWAS) conducted in European and Chinese populations identified interesting candidate genes, including *ACSL5*, which may link genetic susceptibility to metabolic regulation and body weight [123]. Another intriguing line of investigation has explored potential parallels between severe weight loss in ALS and cancer cachexia. Some studies have attempted to clarify whether shared biological mechanisms may contribute to both conditions. Although progress remains limited, experimental work in animal models has begun to explore possible mechanistic connections between cancer-related cachexia and severe weight loss in ALS [124].

### 5.2. Hypermetabolic State

Bouteloup and colleagues [125] measured resting energy expenditure (REE) in 61 individuals with ALS and found that 48% exhibited measured REE approximately 20% higher than the calculated mean REE. Importantly, this hypermetabolism persisted even when measured REE was normalized to fat-free mass (FFM). Furthermore, the hypermetabolic state remained relatively stable throughout the course of the disease despite ongoing clinical progression. Although the origin of hypermetabolism remains uncertain, the authors suggested that accumulating evidence points to mitochondrial dysfunction as a possible contributing factor. Subsequent studies involving larger cohorts confirmed these findings. In a study including 315 individuals with ALS, more than half were found to be hypermetabolic, and those with hypermetabolism exceeding 20% above the predicted REE had a significantly worse prognosis than those without hypermetabolism [126,127]. The association between hypermetabolism, faster functional decline, and shorter survival has also been confirmed by other research groups [128,129]. Similar findings have been reported in fALS, as a study of ten individuals with non-SOD1 familial ALS demonstrated hypermetabolism comparable to that observed in sALS [130].

There are several confounders, including disease subtypes (e.g., bulbar, spinal, upper motor neuron–dominant, or lower motor neuron–dominant types), concurrent medications (e.g., corticosteroids, riluzole), and comorbid conditions (e.g., thyroid dysfunction), all of which may independently influence energy expenditure and survival but are rarely accounted for in prior hypermetabolism studies. Therefore, further studies are clearly needed to better understand hypermetabolism in ALS.

An intriguing observation was reported by Nakamura and colleagues [131], who found that hypermetabolism was associated with shorter survival among patients with normal body weight but paradoxically with longer survival among those who were already malnourished. To explain this finding, the authors proposed a new metabolic index incorporating both energy expenditure and nutritional status. These observations highlight the complex relationship between energy metabolism, nutritional status, and disease progression in ALS. Further investigation is needed to clarify the biological mechanisms and clinical significance of hypermetabolism in ALS [132,133].

### 5.3. Lipid Metabolism

Dupuis and colleagues [51] were the first to report that hyperlipidemia was a significant prognostic factor for survival in a large cohort of individuals with ALS. This positive relationship between dyslipidemia and improved survival was subsequently confirmed in a study involving an even larger cohort of ALS patients [134]. Further investigations also explored the prognostic significance of different lipoprotein subclasses, highlighting the potential importance of cholesterol fractions in ALS progression [135].

However, not all studies have reached the same conclusion, and the findings remain somewhat inconsistent. In a prospective observational cohort of 512 individuals with ALS, none of the lipid parameters showed statistically significant associations with survival after adjustment for prognostic covariates [136]. In contrast, analysis of a large ALS registry in southern Germany reported that higher levels of high-density lipoprotein cholesterol (HDL-C) and low-density lipoprotein cholesterol (LDL-C) were associated with higher mortality, whereas higher triglyceride levels were associated with lower mortality [137]. Similarly, a meta-analysis combined with a population-based study found that increased HDL-C levels were associated with poorer survival [138]. Additional studies have examined specific lipid species. In a large prospective clinical cohort, higher levels of α-linolenic acid (ALA) were associated with longer survival and slower functional decline in ALS patients [139]. LDL cholesterol appears to function as an important mediator of ALA transport and metabolism [140]. These findings illustrate the complex relationship between lipid metabolism and ALS progression. Differences in study populations, including age distribution and comorbidities, may partly account for these variable findings.

#### 5.3.1. Preclinical Changes in Lipid Levels

Evidence suggests that lipid abnormalities may precede the clinical onset of ALS. Using Swedish national healthcare data, Mariosa and colleagues [141] reported that during the ten years preceding ALS diagnosis, individuals who later developed ALS showed progressively increasing levels of glucose, LDL-C, HDL-C, apolipoprotein B, and apolipoprotein A-1. Similarly, a large population-based study in England found that HDL, apolipoprotein A1, and LDL levels were associated with ALS risk, suggesting the presence of a preclinical metabolic landscape [142]. Another investigation reported that higher pre-diagnostic HDL-C levels were associated with an increased risk of ALS [143]. In addition, genetically predicted increases in LDL-C levels were significantly associated with a higher risk of ALS [140].

A meta-analysis combined with Mendelian randomization further confirmed associations between LDL-C and total cholesterol with ALS risk, as well as a relationship between apolipoprotein B and the risk of both ALS and frontotemporal dementia [144]. Long-term population data from Norwegian cardiovascular health surveys conducted between 1974 and 2003 demonstrated that higher LDL-C levels were associated with increased ALS risk even more than 40 years later, supporting a potential causal relationship. At the same time, the temporal relationship between triglycerides, total cholesterol, and ALS risk suggests that increases in some lipid biomarkers may also represent metabolic consequences of the disease [145]. Further investigation is needed to find any relationship between such higher lipid levels and the frequency of myocardial infarction.

#### 5.3.2. Cholesterol: Essential but Harmful in Excess

Cholesterol is a central determinant of membrane architecture, signaling, and cellular homeostasis in the central nervous system [146]. Cholesterol also plays an important structural role in specialized microdomains known as lipid rafts. These dynamic multimolecular lipid–protein complexes are enriched in cholesterol, sphingolipids, and signaling proteins and serve as platforms for key signal transduction pathways. Destabilization of the lipid raft structure may alter their biophysical properties and disrupt cellular signaling in ALS [147]. As neurons degenerate, cholesterol is released within the CNS, leading to the generation of more than 40 different sterols and cholesterol metabolites. However, the enzymatic pathways responsible for catabolizing these sterols appear to be impaired in ALS, potentially allowing toxic cholesterol derivatives to accumulate within the CNS [148]. Furthermore, the accumulation of cholesterol in mitochondria above the physiological levels negatively impacts mitochondrial function [149]. Similarly, abnormal cholesterol metabolism has also been demonstrated in skeletal muscle. Myotubes derived from muscle biopsies of individuals with ALS show excessive cholesterol accumulation, accompanied by activation of the lysosomal cholesterol transporters NPC1 and NPC2. These muscle cells appear to compensate metabolically by shifting toward the preferential utilization of fatty acids. Notably, this cholesterol dysregulation has been observed not only in muscle samples from symptomatic ALS patients, but also in presymptomatic carriers of ALS-associated mutations [150].

#### 5.3.3. Other Lipid Alterations

Additional lipid-related abnormalities have been identified in ALS. Alterations in the gut microbiome have been associated with distinct plasma lipid profiles in ALS patients, suggesting interactions between microbial composition and systemic lipid metabolism [151]. Genetic factors may also influence lipid-related pathways in ALS. For example, the apolipoprotein E (APOE) genotype—particularly the ε2 allele—has been reported as a potential risk factor for cognitive impairment in ALS [152]. Metabolomic studies using Mendelian randomization approaches have identified abnormalities in carnitine metabolism and short chain acylcarnitine pathways, suggesting impaired fatty acid oxidation and mitochondrial lipid metabolism in ALS [153,154]. Lipidomic analyses further demonstrate the complexity of lipid alterations in ALS. More than 390 distinct lipid species have been identified in plasma and serum samples from ALS patients. Large numbers of lipid species have also been detected in skin-derived fibroblasts and in mitochondrial and endoplasmic reticulum fractions. These findings suggest that lipidomic profiling may provide important insights into metabolic alterations in ALS and may represent a promising direction for future research [155,156,157,158,159].

### 5.4. Carbohydrate and Glucose Metabolism

Abnormalities in glucose metabolism in ALS were first reported in 1964 [160]. Insulin resistance in ALS was also described several years later [161]. A meta-analysis examining this issue concluded that the risk of developing ALS was significantly lower among individuals with diabetes mellitus than among those without diabetes [162]. However, a systematic review found no consistent association between diabetes and ALS severity, progression, or survival [163]. A large population-based study conducted in Turin, Italy, also suggested a protective association between diabetes and ALS risk [164]. Glucose metabolism presents important challenges for understanding ALS pathophysiology. ALS patients frequently exhibit impaired glucose tolerance, which may be associated with elevated levels of free fatty acids (FFAs), potentially contributing to insulin resistance as part of a multifactorial metabolic disturbance [165]. At the cellular level, ALS muscle cells demonstrate metabolic reprogramming characterized by reduced glycolysis and increased reliance on fatty acid utilization. In contrast, ALS motor neurons appear to increase glycolysis as a compensatory response to impaired mitochondrial function and reduced lipid β-oxidation [166]. These findings highlight the complex and cell-type-specific metabolic alterations occurring in ALS. Metabolic differences may also exist between clinical subtypes of ALS. One study suggested that individuals with spinal-onset ALS exhibit greater peripheral insulin resistance than those with bulbar-onset disease, potentially reflecting more extensive skeletal muscle involvement [167].

Dietary factors may also influence ALS progression. In a large prospective ALS cohort, dietary macronutrient intake was examined to determine whether specific nutrients affected disease progression and survival. Foods with higher dietary glycemic index and glycemic load—reflecting greater glucose availability—were associated with slower disease progression [168]. Interestingly, subsequent analyses indicated that this beneficial effect was observed primarily among ALS patients receiving riluzole treatment [169]. Notably, riluzole, one of the few approved disease-modifying therapies for ALS, has been reported to improve cerebral glucose metabolism and overall nutritional status [118,170,171]. The precise mechanisms underlying these metabolic effects require further detailed investigation, the results of which would be highly beneficial.

### 5.5. Amino Acid Metabolism

Amino acid synthesis and interconversion in both skeletal muscle (e.g., alanine and glutamine) and the brain, including neuronal and astrocytic crosstalk (e.g., glutamate–glutamine cycling), depend fundamentally on mitochondrial tricarboxylic acid (TCA) cycle flux and cellular redox balance, processes largely coordinated through hepatic metabolism. Consequently, mitochondrial dysfunction can directly perturb local neurotransmitter homeostasis as well as systemic nitrogen trafficking under pathological conditions [172,173,174,175].

**Figure 1:**
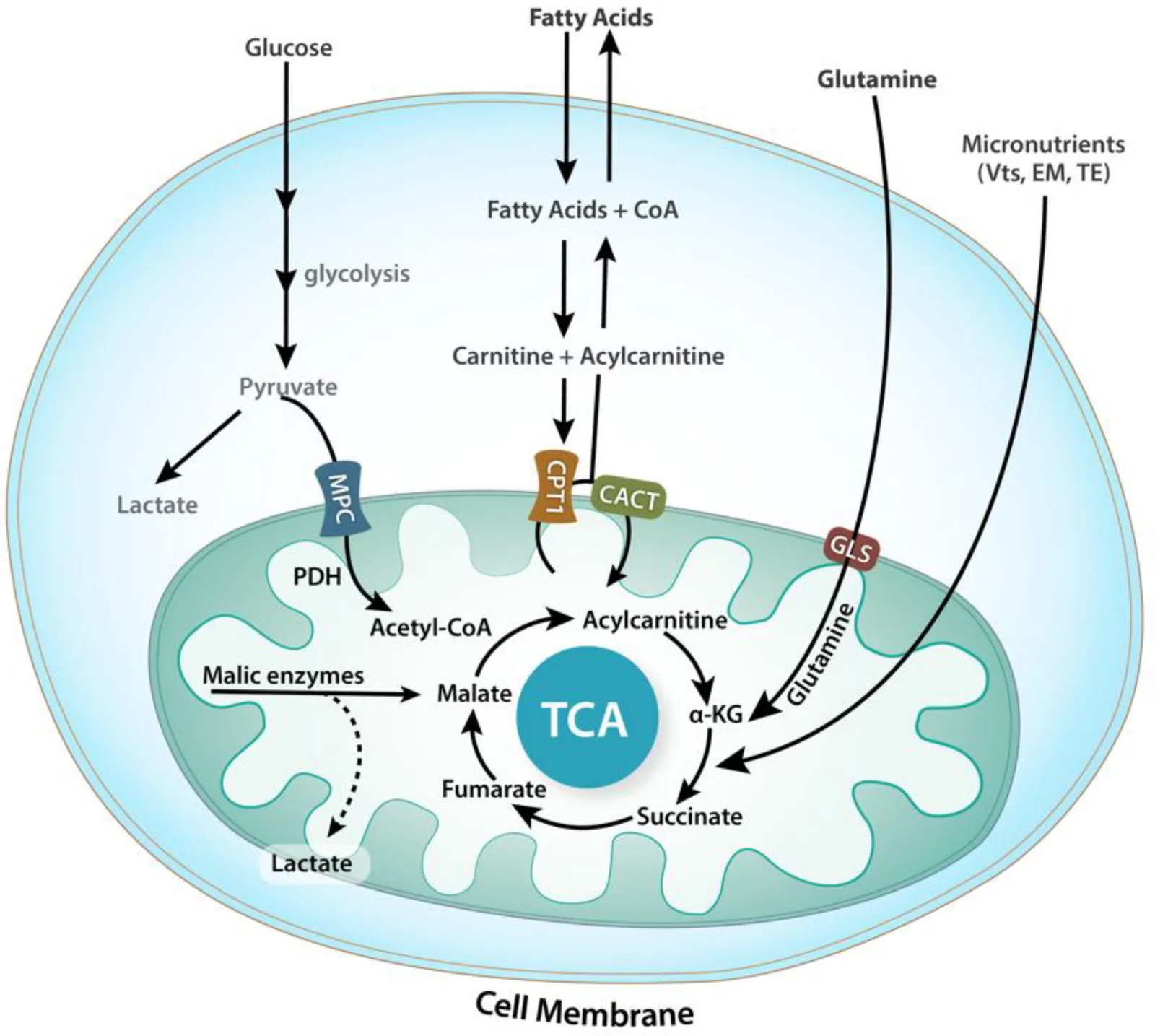
Simplified schematic representation of the major metabolic pathways contributing to neuronal mitochondrial energy metabolism. Glucose-derived pyruvate enters mitochondria through the mitochondrial pyruvate carrier (MPC) and is converted by pyruvate dehydrogenase (PDH) to acetyl-CoA, which enters the tricarboxylic acid (TCA) cycle. Fatty acids and glutamine provide alternative substrates for mitochondrial metabolism, particularly when glucose utilization is impaired. Micronutrients support mitochondrial metabolism through specific biochemical functions: vitamin-derived cofactors participate in pyruvate metabolism, the TCA cycle, and oxidative phosphorylation, whereas essential minerals and trace elements serve as enzyme cofactors supporting respiratory-chain function and redox homeostasis. Other vitamins contribute to antioxidant defense, membrane integrity, calcium signaling, and bioenergetic regulation. This figure is intended as a simplified conceptual overview and does not depict every individual micronutrient-dependent biochemical reaction. Abbreviations: α-KG, α-ketoglutarate; CACT, carnitine-acylcarnitine translocase; CPTI, carnitine palmitoyltransferase I; EM, essential minerals; GLS, glutaminase; MPC, mitochondrial pyruvate carrier; PDH, pyruvate dehydrogenase; TCA, tricarboxylic acid cycle; TE, trace elements; Vts, vitamins.

Alterations in amino acid metabolism in ALS have been investigated in multiple metabolomic studies [176,177,178,179,180,181,182]. Many of these studies report the dysregulation of glutamate, the principal excitatory neurotransmitter, with elevated glutamate levels frequently associated with disease progression [180]. Arginine has also attracted interest as a potentially important amino acid in ALS. Altered arginine metabolism may contribute not only to neurodegeneration, but also to clinical manifestations such as muscle cramps [178,181,183]. In addition, combined alterations in arginine and proline levels have been associated with disease progression [184]. Another metabolomic investigation reported lower leucine levels together with elevated glutamate concentrations in individuals with early-onset ALS compared with matched controls [185]. Overall, however, a comprehensive discussion of amino acid metabolism in ALS was beyond the scope of the present review. Nevertheless, glutamate metabolism is known to be closely linked to mitochondrial function, particularly through oxidative deamination and the entry of glutamate-derived carbon into the tricarboxylic acid (TCA) cycle [186,187] (see Figure 1).

Metabolomics provides a powerful approach for analyzing large numbers of metabolites simultaneously and may offer valuable insights into ALS pathophysiology. Longitudinal metabolomic studies examining amino acids and related metabolic pathways across disease stages, clinical subtypes, and multiple tissues or cell types derived from individuals with ALS may further clarify the role of amino acid metabolism in disease mechanisms. Because variability may arise from differences in disease stage, tissue specificity, and methodology, there is a need for standardized, multi-omic approaches to resolve these discrepancies [159,181,188].

### 5.6. Micronutrient Metabolism

Micronutrients include essential minerals, trace elements (or trace metals), and vitamins, all of which play indispensable roles in cellular and mitochondrial metabolism throughout the body [189]. Table 3 and Table 4 summarize the essential minerals and trace elements. Remarkably, most minerals currently recognized as essential for human health participate directly or indirectly in mitochondrial physiology, where they function as enzyme cofactors, structural components of respiratory complexes, and regulators of oxidative phosphorylation, calcium signaling, antioxidant defense, and redox homeostasis [190,191,192]. Unlike minerals, which primarily serve as enzyme cofactors or structural components, most vitamins participate in mitochondrial metabolism as coenzymes, antioxidants, or regulators of mitochondrial signaling. Notably, all eight B vitamins are directly involved in mitochondrial metabolic pathways, whereas vitamins C, D, E, and K contribute to mitochondrial redox homeostasis, membrane integrity, calcium signaling, and bioenergetic regulation [193,194] (see also Figure 1).

Among the trace elements, iron has recently received increasing attention in ALS research because of its central role in mitochondrial metabolism and oxidative injury [195,196]. Ferroptosis, a regulated form of cell death driven by iron-dependent lipid peroxidation, is now recognized as an important contributor to the pathogenesis of several neurodegenerative diseases, including ALS [197]. Accordingly, iron chelation therapy with deferiprone has been evaluated in clinical trials involving individuals with ALS [183].

Among the vitamins, vitamin E and vitamin B_12_ are particularly relevant to mitochondrial metabolism in ALS. Vitamin E functions as a lipid-soluble antioxidant within mitochondrial membranes, protecting them against oxidative damage [198], whereas vitamin B_12_ has been shown to reduce TDP-43 toxicity by alleviating oxidative stress and mitochondrial dysfunction [199]. Methylcobalamin, the biologically active form of vitamin B_12_, has also been evaluated at pharmacological doses as a therapeutic agent for ALS [200].

In addition to essential micronutrients, several naturally occurring compounds support mitochondrial function, including coenzyme Q10, L-carnitine (particularly acetyl-L-carnitine), α-lipoic acid, creatine, taurine, choline, polyamines (particularly spermidine), and N-acetylcysteine through its role in glutathione metabolism. Although these compounds are not classified as essential micronutrients, they are considered mitochondrial bioactive compounds or mitochondrial metabolic cofactors [201].

Collectively, these findings underscore the close relationship between micronutrients and mitochondrial metabolism and further support the concept that optimizing micronutrient homeostasis may represent a complementary therapeutic strategy for ALS.

### 5.7. Summary of Metabolism Section

Collectively, abnormalities in lipid, glucose, amino acid metabolism and micronutrients converge on mitochondrial energy production and the processing of metabolites, suggesting that mitochondrial dysfunction may represent a central mechanistic link underlying metabolic disturbances in ALS. A simplified relationship is shown in Figure 1.

## 6. ALS Is a Mitochondrial Disease

DiMauro [202] emphasized the emergence of a distinct conceptual framework of mitochondrial genetics, separate from classical Mendelian genetics, based on three defining features of mitochondrial DNA (mtDNA): (1) polyplasmy, referring to the presence of many copies of the mitochondrial genome within each cell; (2) maternal inheritance; and (3) mitotic segregation. Mitochondria respond to both intrinsic and extrinsic stresses and influence cellular and organismal function through metabolic signaling within cells and between distant tissues. In this sense, mitochondria occupy a central position at the intersection of catabolic and anabolic metabolism, representing a fundamental hub of cellular energy regulation [203]. However, an important distinction exists between ALS and primary mitochondrial diseases. Extraocular muscle involvement, including external ophthalmoplegia, is a characteristic manifestation of several primary mitochondrial disorders, whereas the motor neurons controlling extraocular movements and the extraocular muscles themselves are mostly preserved in ALS. This striking contrast suggests fundamental differences in the nature and tissue selectivity of mitochondrial dysfunction between ALS and primary mitochondrial diseases.

Gorman and colleagues [204] further noted that mutations affecting mitochondrial function may arise through multiple mechanisms, including sporadic mutations, maternally inherited mutations, and mutations following Mendelian inheritance patterns. Mitochondrial DNA (mtDNA) abnormalities include deletions, duplications, inversions, point mutations, and depletion of mtDNA copy number. Because mitochondria play essential roles in numerous cellular processes, such mutations can produce a broad spectrum of disease manifestations. Moreover, because each cell contains hundreds to thousands of mtDNA copies, the proportion of mutant mtDNA—referred to as heteroplasmy—can profoundly influence cellular and clinical phenotypes [204]. Despite these distinctive genetic features, ALS does not exhibit the classical maternal inheritance pattern typical of primary mitochondrial diseases. Familial ALS most commonly follows an autosomal dominant inheritance pattern [205].

Mitochondrial genetics is a rapidly expanding field that may provide important insights into ALS pathogenesis. An early report described a heteroplasmic 5-bp microdeletion at the 5′ end of the mitochondrial cytochrome c oxidase subunit I (MT-CO1) gene in association with motor neuron disease [206]. More recently, rare loss-of-function variants in ACADM and DNA2, together with reduced expression of these genes in motor neurons, were associated with an approximately 50% shorter survival in ALS; both genes encode proteins involved in mitochondrial function [207]. Circulating cell-free mitochondrial DNA (mtDNA) has also been investigated as a potential biomarker of mitochondrial injury, inflammation, and damage-associated molecular signaling in ALS [208]. In addition, variants in several nuclear-encoded mitochondrial genes have recently been implicated in ALS susceptibility and disease pathogenesis [209]. Variants in POLG have likewise been associated with ALS, linking defective mtDNA maintenance, altered expression of mitochondrial pathways, and impaired mitophagy to ALS pathogenesis [210].

Mitochondria exhibit marked tissue-specific heterogeneity in morphology, abundance, metabolic activity, and proteomic composition, reflecting the distinct bioenergetic and physiological requirements of individual tissues. Evidence from humans and mice indicates that aspects of this tissue-specific mitochondrial organization are conserved across mammalian species, including patterns of mitochondrial DNA copy number [211,212]. Studies in a large mammalian model, the water buffalo (Bubalus bubalis), further demonstrated marked tissue-specific differences in mitochondrial bioenergetic activity, oxidative phosphorylation (OXPHOS) enzyme activity, mitochondrial DNA copy number, and mitochondrial protein-coding gene expression [213,214]. Together, these findings across rodents, large mammals, and humans support a broader, evolutionarily conserved mammalian framework of tissue-specific mitochondrial specialization. Such conserved specialization may help explain why mitochondrial dysfunction produces distinct tissue vulnerabilities and may contribute to the multisystem involvement observed in ALS.

### 6.1. Mitochondrial Changes in the CNS and Motor Neurons of Sporadic ALS

One of the most comprehensive ultrastructural studies of anterior horn motor neurons from ALS autopsy cases was performed by Sasaki and Iwata [215]. It demonstrated clear mitochondrial abnormalities. Examination of motor neurons from 14 ALS cases revealed swollen mitochondria with markedly altered cristae and prominent mitochondrial aggregation within neuronal somata. These mitochondria often contain filamentous structures within the inner compartment and multilayered cristae arranged in stacked linear arrays in longitudinal sections. Such abnormalities were not observed in control motor neurons. MR spectroscopy (^31^P-MRS) studies have also suggested mitochondrial dysfunction, particularly reduced oxidative phosphorylation, in the ALS brainstem and in skeletal muscles such as the anterior tibialis [216]. In addition, voxel-based MRI analyses have demonstrated increased focal mitochondrial tissue density in multiple brain regions, including frontal, temporal, cerebellar, opercular, and thalamic areas. These findings suggest that metabolically active brain regions may be particularly vulnerable to degeneration in ALS [28].

Analyses of mtDNA provide further support for mitochondrial involvement. Increased levels of mutant mtDNA have been reported in ALS spinal cord, while citrate synthase activity—a marker of mitochondrial content—is reduced, suggesting mitochondrial loss [217]. Consistent with this, reduced expression of mtDNA-encoded respiratory chain genes has also been observed [218]. Additional studies demonstrated decreased activity of respiratory chain complex IV (COX) in spinal cord gray matter, despite increased mitochondrial density and altered distribution, suggesting compensatory mitochondrial proliferation in response to impaired function [219]. Proteomic analyses further revealed reduced synaptic mitochondrial ATP synthase levels [220]. Epigenetic alterations may also contribute: reduced methylation of the mitochondrial D-loop region has been reported in sporadic ALS and is inversely correlated with mtDNA copy number, suggesting compensatory mitochondrial replication [221]. ALS-associated proteins further disrupt mitochondrial function. TDP-43, a hallmark pathological protein in sporadic ALS, accumulates in axons and interferes with mitochondrial transport, reducing mitochondrial protein content at synaptic terminals [222]. Similarly, FUS disrupts mitochondria–endoplasmic reticulum signaling, impairing mitochondrial function and ATP production [223].

### 6.2. Mitochondrial Changes in Astrocytes

Mitocondria vary in morphology and function across different tissues; however, their diversity among different brain cell types remains incompletely understood [224]. The cellular and functional interactions between neurons and surrounding astrocytes are central to ALS pathogenesis. Compared with astrocytes, neurons possess more extensive mitochondrial networks and organelle interactions, whereas astrocytes contain relatively more lysosomes and lipid droplet interactions and exhibit a less robust organelle response to acute oxidative or endoplasmic reticulum (ER) stress [225]. Astrocytic mitochondria metabolize long-chain fatty acids more efficiently than neuronal mitochondria [224]. Although astrocytes generally have lower mitochondrial respiratory chain activity, they maintain a well-developed mitochondrial network whose morphology and function change during glial reactivity [226]. Astrocytic mitochondria are highly dynamic, undergoing continuous fission and fusion, intracellular trafficking, and selective degradation [227]. Mitochondrial transfer from astrocytes to neurons has been proposed as a neuroprotective mechanism [228], whereas transfer of dysfunctional mitochondria has recently been demonstrated in a Huntington disease mouse model, suggesting that astrocyte-neuron mitochondrial exchange may also contribute to neurodegeneration [229].

Studies in transgenic ALS models have identified aberrant glial cells with enhanced proliferative activity but impaired mitochondrial bioenergetics, indicating metabolic reprogramming associated with motor neuron degeneration [230]. Likewise, expression of mutant SOD1(G93A) in astrocytes alters mitochondrial redox state and decreases mitochondrial membrane potential in neighboring motor neurons, thereby increasing their vulnerability to neurotoxic injury [231]. A unique study of Kii ALS/Parkinsonism-Dementia Complex (ALS/PDC) identified marked dysregulation of CHCHD2 in astrocytes together with ciliary dysfunction, abnormal mitochondrial morphology, and metabolic alterations, suggesting impaired astrocytic metabolic support of neurons [232].

The development of fibroblast reprogramming and human iPSC-derived astrocytes has greatly advanced the investigation of astrocytic dysfunction in ALS. However, direct studies of mitochondrial and energy metabolism in astrocytes derived from individuals with sporadic ALS remain remarkably limited. Allen and colleagues [233] demonstrated loss of metabolic flexibility, including impaired adenosine, fructose, and glycogen metabolism, in astrocytes derived from both sporadic and C9orf72-associated ALS. Additional studies in C9orf72 astrocytes have reported increased mitochondrial DNA single-nucleotide variants, mitochondrial dysfunction, oxidative stress, and reduced metabolic support for motor neurons [234,235,236]. Using human iPSC-derived astrocytes, Zhao et al. further showed that mutant C9orf72 astrocytes impaired motor neuron excitability through reduced voltage-gated Na+ and K+ currents, abnormalities that were reversed by CRISPR/Cas9-mediated excision of the repeat expansion, confirming both cell-autonomous and non-cell-autonomous pathogenic mechanisms [237]. In addition, CuATSM treatment of astrocytes derived from both sporadic and familial ALS cases improved motor neuron survival in an astrocyte–motor neuron co-culture system [238], providing further evidence that metabolic modulation of ALS astrocytes may influence their non-cell-autonomous effects on motor neurons.

Similar mitochondrial abnormalities have been demonstrated in several other familial ALS models. Astrocytes carrying the CCNF^S621G^ mutation exhibit impaired mitochondrial membrane potential, abnormal mitochondrial network morphology, and the suppression of repetitive neuronal firing, supporting an astrocyte-driven mechanism contributing to ALS/FTD pathogenesis [239]. VCP-mutant astrocytes display mitochondrial depolarization, lipid droplet accumulation, and the activation of hypoxic signaling, resulting in impaired neuronal support [240,241]. Likewise, TARDBP-mutant astrocytes show cytoplasmic TDP-43 accumulation, increased polyphosphate levels, and reduced physiological function [242], whereas FUS-mutant astrocytes impair motor neuron neurite outgrowth and neuromuscular junction formation, abnormalities rescued by isogenic control astrocytes [10].

Collectively, these studies demonstrate remarkable advances in our understanding of astrocytic mitochondrial abnormalities, particularly through studies using iPSC-derived astrocytes from familial ALS cases. However, studies directly investigating mitochondrial and energy-metabolic abnormalities in astrocytes from sporadic ALS remain very limited [233,238]. Considering the central role of astrocytes in the non-cell-autonomous mechanisms underlying ALS pathogenesis, such investigations should be substantially expanded in sporadic ALS, which accounts for approximately 90% of all ALS cases.

### 6.3. Mitochondrial Abnormalities in Skeletal Muscle

Mitochondrial abnormalities are also evident in skeletal muscle. In studies of ALS muscle biopsies, histochemically COX-negative fibers were frequently observed, with biochemical evidence of COX deficiency and multiple mtDNA deletions [243], combined with ultrastructural mitochondrial abnormalities [244]. Ultrastructural abnormalities have been reported in approximately 10% of biopsies, including giant mitochondria, paracrystalline inclusions, and abnormal cristae in subsarcolemmal regions [245], demonstrated reduced complex I activity and decreased mtDNA content in approximately half of the ALS muscle samples, suggesting intrinsic mitochondrial defects rather than secondary effects of denervation. Their subsequent study supported the original viewpoint that oxygen radical-induced impairment of mtDNA is of pathophysiological significance in at least a subgroup of patients with sALS [246]. Further studies indicate early global mitochondrial dysfunction in skeletal muscle. Altered mitochondrial enzyme activity—including increased complex II/citrate synthase ratios and decreased lactate dehydrogenase activity—has been observed even in early-stage ALS [247]. However, findings are not entirely consistent. Some studies using skinned muscle fibers in oxygraphic systems reported mitochondrial respiration comparable to sedentary controls [248], and others found no significant differences compared with other denervating disorders [249]. These discrepancies suggest heterogeneity in mitochondrial involvement, possibly related to disease stage or methodology.

Recent work using the low-copy SOD1-G93A mouse [250,251] has further strengthened the concept that skeletal muscle mitochondrial abnormalities evolve in a stage-dependent manner. Ultrastructural analysis of quadriceps muscle demonstrated mosaic degeneration of subsarcolemmal mitochondria during the early symptomatic stage, before widespread structural deterioration. As disease progressed, mitochondrial respiratory capacity, oxidative phosphorylation efficiency, and calcium retention capacity declined, accompanied by increased lipid peroxidation. These findings demonstrate progressive impairment of skeletal muscle mitochondrial bioenergetics and provide further evidence that mitochondrial pathology in ALS extends beyond motor neurons, supporting the systemic nature of the disease. Furthermore, muscle-specific expression of mutant SOD1 induces metabolic remodeling before motor neuron degeneration, suggesting that skeletal muscle may actively contribute to ALS pathogenesis rather than solely representing a secondary target of denervation [252,253]. Collectively, these findings support a potential active contribution of skeletal muscle mitochondrial pathology to disease mechanisms and progression.

### 6.4. Cultured Skin Fibroblasts

Transgenic animal models do not adequately reflect the heterogeneity of ALS. On the other hand, skin-derived fibroblasts from sporadic ALS patients and familial ALS thus provide diversity and a highly useful model for studying mitochondrial function [254,255]. These cells exhibit hypermetabolism with altered bioenergetics. Notably, the lack of correlation between increased metabolic activity and ATP production suggests compensatory responses to inefficient energy utilization. Fibroblasts demonstrate increased uncoupled respiration, elevated mitochondrial membrane potential, increased glycolysis, and a reduced mitochondrial oxidation metabolism (OCR/ECAR ratio), indicating altered mitochondrial bioenergetics [256]. Similar findings of impaired oxidative phosphorylation have been reported in both sporadic and familial ALS, including relatively mild defects in C9orf72-associated ALS [257]. An important question is whether these findings reflect systemic metabolic alterations relevant to disease-relevant cell types. If mitochondrial inefficiency is systemic, high-energy-demanding cells such as motor neurons may be particularly vulnerable. However, fibroblasts do not consistently reproduce hallmark ALS pathology such as TDP-43 aggregation, warranting cautious interpretation [258]. On the other hand, a recent metabo-lipidomics study showed that, despite the absence of mitochondrial respiratory or mtDNA defects, sporadic ALS fibroblasts display robust disease-specific metabolomic (purine, pyrimidine, and energy pathway dysregulation) and lipidomic (mitochondria–ER phosphatidylcholine PC 36:4p alterations) signatures, aligning with systemic ALS metabolic dysfunction and supporting their potential as a translatable, non-neuronal model for mechanistic and biomarker studies [158]. Furthermore, these models including iPSC models (see below), human motor neurons, astrocytes, and even co-culture systems, can be utilized to investigate functions along with pharmaceutical effects in a human cell paradigm [238].

### 6.5. iPSC-Derived Motor Neurons

Induced pluripotent stem cell (iPSC)-derived motor neurons enable the direct investigation of mitochondrial dysfunction in disease-relevant cells. Studies demonstrate impaired communication between mitochondria and the endoplasmic reticulum (ER), disrupting pyruvate utilization and shifting energy metabolism toward fatty acid oxidation [259]. This highlights the importance of mitochondria-associated ER membranes (MAMs) in neuronal metabolism. Cytoplasmic TDP-43 accumulation can also induce mtDNA release upon mitochondrial entry, leading to progressive mitochondrial damage [260]. Additional studies show increased reactive oxygen species production, mitochondrial membrane depolarization, impaired oxidative phosphorylation, reduced ATP production, and defective mitochondrial protein import in ALS motor neurons [261]. These findings collectively demonstrate profound mitochondrial dysfunction in ALS motor neurons derived from patient cells.

### 6.6. Mitochondrial Changes in Sporadic ALS

Table 5 summarizes all the mitochondrial abnormalities across multiple cell types in sporadic ALS, reflecting contributions from non–cell-autonomous toxicity mechanisms [7].

### 6.7. Mitochondrial Changes in Familial ALS

**Figure 2:**
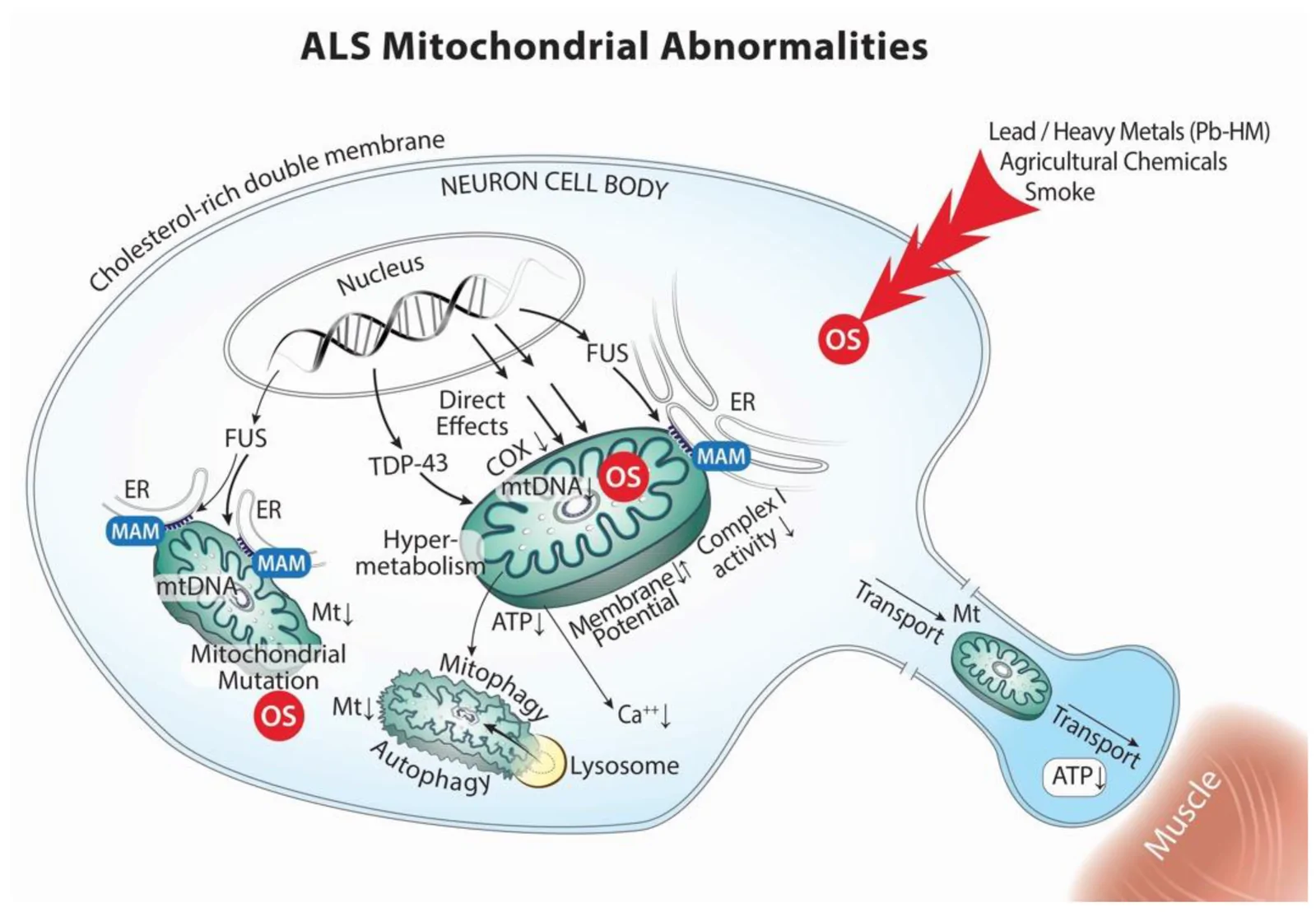
Schematic summary of the mitochondrial abnormalities shared between sporadic ALS (Table 5) and familial ALS (Table 6). Mitochondrial abnormalities reported exclusively in either sporadic or familial ALS are not depicted. Increased intraneuronal Ca^2+^ levels, although not classified as a primary mitochondrial abnormality in the studies summarized in Table 5 and Table 6, are a well-recognized downstream consequence of mitochondrial dysfunction. Similarly, oxidative stress (OS) is a common feature of mitochondrial dysfunction and is widely observed in both mitochondria and neuronal cells in ALS. Abbreviations: ATP = adenosine triphosphate; COX = cytochrome c oxidase; ER = endoplasmic reticulum; FUS = fused in sarcoma; MAM = mitochondria-associated membrane; Mt = mitochondria; OS = oxidative stress; and TDP-43 = TAR DNA-binding protein 43. Red thunder arrow indicates exogenous OS stimuli.

More than 40 genetic mutations have been identified in familial ALS. Table 6 summarizes the most common mutations along with selected rare forms, which would constitute the majority of fALS cases. Recent advances, including direct conversion of fibroblasts into induced motor neurons (iMNs), allow for the investigation of mitochondrial dysfunction without a pluripotent stage [261]. These approaches, together with fibroblast and iPSC-based models, provide powerful platforms to study mitochondrial mechanisms across genetic subtypes of ALS. The accompanying Figure 2 summarizes shared biochemical features of mitochondrial dysfunction in both familial and sporadic ALS.

As seen in Table 6, a spectrum of mitochondrial abnormalities has been observed in these common familial ALS forms and two rare variants, with the exception of mutations of the Angiogenin (ANG) gene, which encodes a neuroprotective ribonuclease and for which no mitochondrial abnormalities have been clearly described [262]. Strikingly, mitochondrial alterations are present across nearly all of these genetic forms. The predominant abnormality is bioenergetic failure, followed by impairments in mitochondrial motility and transport, mitophagy and quality control, mitochondria–ER signaling at the mitochondria-associated membrane (MAMs), and mitochondrial DNA deletions, damage, and mutations. Figure 2 illustrates these shared mitochondrial defects. Furthermore, reviewing the types of mitochondrial abnormalities observed in sALS and fALS (Table 5 and Table 6), there are again striking similarities in these abnormalities.

### 6.8. Other Mitochondrial Abnormalities

In ALS, structurally and functionally abnormal mitochondria—including those with swelling, cristae disruption, impaired dynamics, and defective transport—accumulate due to impaired mitophagy. The persistence of these dysfunctional mitochondria leads to increased reactive oxygen species production, reduced ATP generation, and progressive cellular injury. This self-amplifying cycle of mitochondrial damage is likely a central driver of motor neuron degeneration [286,287]. Calcium buffering is one of the crucial functions of mitochondria, as intracellular calcium levels must be tightly regulated to control multiple cellular processes [288]. More recently, particular attention has been directed toward the signaling interface for physical and functional interactions between mitochondria and the ER, known as the mitochondria-associated membrane (MAM), which is crucial for cellular homeostasis. These two organelles are intimately connected and collaborate in essential processes, such as calcium homeostasis and phospholipid biosynthesis [289].

## 7. Oxidative Stress

Oxidative stress (OS) refers to a pathological condition characterized by increased levels of reactive oxygen species (ROS) and reactive nitrogen species (RNS). These reactive molecules arise from endogenous sources—primarily mitochondria and NADPH oxidases—as well as extensive exogenous sources (see below). Although the pathogenesis of chronic neurodegenerative diseases, including ALS, is widely considered multifactorial, oxidative stress is often regarded as a common underlying mechanism [290]. Accordingly, oxidative stress has been proposed as a major contributor to ALS pathophysiology [291]. In this section, we review evidence supporting the involvement of oxidative stress in ALS.

Postmortem studies have provided early and consistent evidence of oxidative damage in ALS. Analysis of frontal cortex tissue from individuals with sporadic ALS demonstrated markedly elevated levels of protein carbonyls—oxidized protein products—as well as 8-hydroxy-2′-deoxyguanosine (8-OHdG), a marker of oxidative DNA damage [292]. Immunohistochemical studies have also shown increased lipid peroxidation and protein glycoxidation in spinal cord motor neurons and glial cells from ALS autopsy samples [293]. These findings are widely interpreted as evidence of oxidative stress in ALS. Further postmortem analyses suggest interactions between oxidative stress and neuronal vulnerability. In a study of spinal cord tissue from ALS cases, surviving motor neurons demonstrated upregulated redox-related responses, implying that neurons with more robust antioxidant defenses may be relatively protected [294]. Another study reported that protein oxidative damage in ALS spinal cord tissue was closely associated with alterations in fatty acid composition and dysfunction of mitochondrial respiratory chain complexes I and III, supporting a mechanistic link between mitochondrial impairment and oxidative stress [295]. Inflammatory reactions to OS appear to be additional contributors to disease progression [296].

In vivo imaging studies also support the presence of oxidative stress in ALS. The PET tracer diacetyl-bis (N4-methylthiosemicarbazone) labeled with copper (Cu-ATSM) accumulates in cells under reductive intracellular conditions, particularly when mitochondrial electron transport chain activity is impaired [297]. Increased Cu-ATSM uptake has been observed in cortical regions surrounding the central sulcus, including the motor cortex, suggesting altered redox states in ALS [297]. Biochemical analyses of biological fluids further support oxidative stress in ALS. Elevated levels of hydroxyl radicals in blood, and increased concentrations of ascorbate free radicals and 8-oxodG in CSF have been reported in both sporadic and familial ALS [298]. Oxidized coenzyme Q_10_ levels in CSF are also increased and inversely correlated with disease duration, suggesting progressive mitochondrial oxidative damage [299]. Additional studies have demonstrated increased oxidation of total thiol (-SH) groups, elevated nitric oxide levels, and reduced superoxide dismutase activity in CSF, indicating an imbalance between oxidant production and antioxidant defenses [300,301].

Evidence of increased oxidative stress, reflected by elevated cerebrospinal fluid (CSF) nitrotyrosine levels, was significantly reduced following six months of intravenous edaravone treatment in patients with ALS [302]. Based on these findings, a subsequent controlled clinical trial demonstrated that edaravone slowed disease progression as measured by the ALSFRS-R [303]. Nevertheless, the effectiveness of edaravone remains uncertain, as a subsequent European study reported less favorable results. Although findings in blood and erythrocytes have been less consistent, urinary biomarkers such as 8-oxodG and 15-F2t-isoprostane—markers of oxidative damage and lipid peroxidation—are significantly elevated in individuals with ALS [304].

Experimental studies using cultured fibroblasts from sporadic and familial ALS patients provide additional insight. Chronic exposure to coenzyme Q10 partially restored impaired oxidative phosphorylation in these cells [257]. In another model, fibroblasts exposed to hydrogen peroxide (H_2_O_2_) demonstrated increased vulnerability to oxidative injury, particularly in SOD1-associated familial ALS, while fibroblasts from sporadic ALS patients also showed greater susceptibility than those from healthy controls [305].

### Exogenous Oxidative Stress

Exogenous sources of oxidative stress may further exacerbate endogenous oxidative damage in ALS, including radiation, certain drugs, dietary factors, cigarette smoking, and environmental pollutants implicated as potential contributors, including heavy metals, agricultural chemicals, and other toxins [306]. Current evidence supports a model in which ALS arises from interactions between genetic susceptibility and environmental exposures that promote oxidative stress and neurodegeneration [307]. Importantly, environmental influences are unlikely to act through acute toxicity alone. Rather, cumulative exposures over many years may gradually increase neuronal vulnerability and contribute to disease onset [308]. Recent case–control studies continue to identify previously recognized environmental risk factors, reinforcing the role of environmental oxidative stressors including heavy metals and agricultural chemicals [309,310].

## 8. Future Perspectives for Mitochondrial Therapeutics in ALS

Since riluzole was approved in 1995, it is estimated that more than 100 clinical trials have been conducted in ALS, and the number of trials has increased rapidly over time and is expected to continue to grow [311,312,313]. Most therapeutic strategies have been developed on the basis of biologically plausible hypotheses derived from experimental studies, despite the fact that the precise pathogenesis of ALS remains incompletely understood. As our understanding of ALS biology continues to advance, future therapeutic candidates are increasingly expected to be guided by more robust mechanistic evidence. It is noteworthy that many therapeutic agents investigated for ALS have targeted mitochondrial dysfunction (Table 7). More importantly, two of the 13 mitochondria-targeted therapies, excluding riluzole, have demonstrated beneficial effects in slowing ALS disease progression. Edaravone has been approved by the U.S. Food and Drug Administration (FDA), whereas methylcobalamin has been approved by the Pharmaceuticals and Medical Devices Agency (PMDA) in Japan. In contrast, vitamin B12 preparations, including methylcobalamin, do not require regulatory approval in the United States or the European Union because they are classified as dietary supplements. Another mitochondria-targeted therapy, AMX0035 (sodium phenylbutyrate plus taurursodiol), showed promising results in an initial clinical trial, suggesting a slowing of disease progression. However, a subsequent pivotal clinical trial failed to confirm these earlier findings. This experience illustrates the challenges and unpredictability of clinical trials in ALS.

Table 8 summarizes the major functional domains of mitochondrial biology together with their corresponding potential therapeutic targets [321,322,323,324,325,326]. Importantly, most of these potential targets are supported by established experimental evidence. Table 8 highlights both the complexity of mitochondrial biology and the broad range of opportunities for future therapeutic development [327]. Furthermore, when mitochondrial dysfunction in astrocytes is also considered, the therapeutic landscape expands even further [238]. Among the many potential targets for mitochondrial protection, potential drugs have already started being investigated [328]. Collectively, these observations suggest that mitochondria-targeted therapy represents a particularly promising and fertile area for future drug development in ALS.

## 9. Discussion

The present review supports a fundamental reframing of ALS as a systemic mitochondrial disease rather than a disorder confined to motor neurons. While the dominant clinical phenotype arises from the degeneration of upper and lower motor neurons, involvement clearly extends to the frontotemporal system, hypothalamus, and additional regions such as the visual/retinal and olfactory systems. Beyond the CNS, evidence increasingly indicates that multiple peripheral organs are also affected, often in a subclinical manner. The breadth of this involvement suggests that ALS is not a focal neurodegenerative disorder but a disease process affecting the entire organism.

A key question is why widespread organ involvement produces relatively limited non-neurological symptoms. One plausible explanation is that most non-neuronal tissues retain mitotic capacity and can compensate for cellular injury through continuous turnover and replacement [329,330]. In contrast, motor neurons and other involved neurons, which are post-mitotic and highly metabolically demanding, lack this compensatory capacity and are therefore selectively vulnerable. This distinction provides a biological framework linking systemic pathology to selective clinical expression.

Accumulating evidence indicates that ALS begins in a long presymptomatic phase before clinical onset. Systemic metabolic alterations—including weight loss, lipid abnormalities, and retinal atrophy changes—can precede diagnosis by years or even decades. Strikingly, elevated LDL-C levels have been associated with increased ALS risk more than 40 years prior to symptom onset, and pathological protein changes such as TDP-43 aggregation in the skin may be detectable decades before clinical disease [46,145]. In parallel, neurofilament biomarkers demonstrate that neuronal injury begins well before symptom onset [331,332,333]. Together, these findings strongly support a prolonged preclinical phase characterized by systemic and metabolic dysregulation [307].

Within this framework, hypermetabolism emerges as one of the central metabolic features of ALS. While increased energy expenditure is partly explained by motor neuron-related phenomena such as spasticity, weakness, and fasciculations, these factors alone are unlikely to account for the full magnitude of metabolic disturbance [116]. A broader explanation involves increased cellular turnover across multiple organ systems, coupled with chronic immune activation and inflammatory responses. These processes require sustained energy input and may substantially contribute to the hypermetabolic state. Further appropriate studies are clearly needed to fully elucidate the mechanisms underlying hypermetabolism in ALS.

As stated earlier, lipid metabolism and composition are highly complex. HDL- and LDL-cholesterol participate in a bidirectional regulatory axis linking hepatic mitochondrial function with systemic lipid metabolism and transport [334,335]. Our review (see above) indicates that a large proportion of individuals with ALS have non-alcoholic fatty liver (steatosis) and mild impairment of liver function [49,50,336,337], which are considered subclinical and may reflect multisystem organ involvement, as observed in other tissues. However, given that most lipids, including HDL and LDL, are synthesized in the liver, we cannot exclude the possibility that this ‘subclinical’ non-alcoholic fatty liver, which occurs in the majority of individuals with ALS, may influence the lipoprotein profiles reported in ALS (see above).

Glucose metabolism also appears tightly coupled to mitochondrial function, and accumulating evidence indicates that this relationship is disrupted in ALS. Impaired mitochondrial oxidative metabolism, including reduced pyruvate dehydrogenase activity and electron transport chain dysfunction, limits the efficient utilization of glucose-derived substrates. As a result, cells shift toward glycolysis with increased lactate production, reflecting metabolic reprogramming (see Figure 1). In parallel, disruption of astrocyte–neuron metabolic coupling further compromises neuronal energy homeostasis. Together, these findings support the concept that ALS is characterized by mitochondrial dysfunction-driven metabolic inflexibility [336,338]. Nevertheless, individuals with ALS may still exhibit functional stabilization in response to hyperglycemic foods, particularly when riluzole is administered concurrently, although the underlying mechanisms remain to be elucidated [183,198].

The mechanisms underlying the lower risk of developing ALS associated with type 2 diabetes, as discussed earlier [162,164], and the observation that high-glycemic foods may slow ALS disease progression [168] remain incompletely understood. In contrast, no consistent association between type 1 diabetes and ALS risk has been established. Importantly, ALS itself does not appear to progress more slowly in individuals with diabetes, although obesity and altered energy metabolism may influence disease progression [339]. Given that the current literature on the relationship between diabetes and ALS remains limited and somewhat fragmentary, further studies are needed to elucidate the biological mechanisms underlying these intriguing observations.

Similar to glucose and lipid metabolism, amino acid (AA) metabolism in ALS is equally complex and profoundly dysregulated. When mitochondrial function is impaired, amino acid metabolism is rerouted rather than simply reduced: entry of amino acid-derived carbon into the TCA cycle declines, leading to the accumulation of intermediates (e.g., alanine, glutamate, and BCAAs) and diversion toward cytosolic pathways such as lactate production and lipid synthesis (see Figure 1). At the same time, nitrogen handling becomes increasingly dependent on glutamine and alanine as buffering systems, while impaired mitochondrial generation of aspartate and disruption of redox balance (elevated NADH/NAD^+^) further constrain biosynthesis and transamination reactions. Collectively, this reflects a loss of metabolic flexibility, with diminished oxidative utilization of amino acids and a shift toward compensatory, non-mitochondrial pathways [340,341].

Mitochondria occupy a central position in this model. As the primary regulators of cellular energy production, redox balance, and metabolic signaling, mitochondrial dysfunction provides a unifying mechanism linking neuronal degeneration with systemic metabolic abnormalities (Figure 2). Importantly, mitochondrial abnormalities are not confined to motor neurons. This review shows that structural and functional mitochondrial defects are present in multiple peripheral tissues, including skeletal muscle, liver, skin, blood cells, and other organs (Table 2), indicating that mitochondrial dysfunction is systemic in ALS. The relative preservation of function in non-neuronal tissues likely reflects differences in metabolic demand and regenerative capacity rather than the absence of pathology. Neurons—particularly motor, frontotemporal, and hypothalamic populations—are uniquely vulnerable due to their exceptionally high energy demands and reliance on highly efficient mitochondrial function. When mitochondrial bioenergetics fail, these cells cannot maintain homeostasis and undergo degeneration. As noted earlier (see above), however, there are fundamental differences between mitochondrial dysfunction in ALS and that observed in primary mitochondrial diseases, as exemplified by the characteristic sparing of ocular muscles in ALS.

Oxidative stress is closely integrated with mitochondrial dysfunction and further amplifies cellular injury. Impaired mitochondrial respiration increases the generation of reactive oxygen species, while reduced antioxidant defenses exacerbate oxidative damage to proteins, lipids, and DNA. This creates a self-reinforcing cycle in which mitochondrial dysfunction drives oxidative stress, and oxidative stress further impairs mitochondrial function. Such a cycle is likely a key driver of progressive cellular degeneration in ALS.

The convergence of evidence from sALS and fALS further strengthens this framework. Despite differences in genetic origin, both forms demonstrate remarkably similar mitochondrial biochemical abnormalities, particularly in bioenergetics, mitophagy and transport. Many ALS-associated gene mutations directly or indirectly impair mitochondrial function, suggesting that mitochondrial dysfunction represents a final common pathway. The traditional distinction between sALS and fALS is therefore increasingly blurred, with shared downstream mechanisms converging on mitochondrial failure. At the same time, these observations provide an opportunity to investigate the pathogenesis of sALS by exploring upstream mechanisms reflected in mitochondrial abnormalities.

From a pathogenic perspective, these observations suggest that ALS may originate as a systemic disorder of mitochondrial and metabolic regulation, with motor neuron degeneration representing the most clinically visible consequence. This perspective shifts the focus from neuron-specific mechanisms to organism-wide bioenergetic failure.

Clinically, this integrated model is consistent with current management strategies. Approaches such as maintaining body weight, supporting energy metabolism, managing lipid profiles, and minimizing oxidative stress [306,342,343], as well as utilizing agents such as riluzole and edaravone, align with the concept of preserving metabolic and mitochondrial function and reducing metabolic stress. While these interventions are not curative, they may partially mitigate downstream consequences of mitochondrial dysfunction.

## 10. Conclusions

The evidence reviewed here supports a unified model in which multisystem involvement, metabolic failure, hypermetabolism, mitochondrial dysfunction, and oxidative stress are not independent processes but tightly interconnected components of ALS pathogenesis. Among these, mitochondrial dysfunction appears to occupy a central and potentially early initiating role. Accordingly, greater emphasis on mitochondrial mechanisms may be critical for advancing therapeutic development. Recognizing ALS as a systemic mitochondrial disease provides a coherent framework that integrates diverse clinical and experimental observations and may guide future research toward identifying upstream mechanisms. To effectively accomplish this task, a concerted effort involving not only neurologists, neurogeneticists, neuroscientists, and neurobiologists but also experts in metabolism and mitochondrial biology will be required. It is hoped that this review will stimulate further research and contribute to the development of more effective disease-modifying therapies in the future.

## Figures and Tables

**Table 1:** Neuronal populations affected in ALS compared with other cortical neurons.

Feature	Motor Neurons	Frontotemporal Neurons	Hypothalamic Neurons	Interneurons	Primary Sensory Neurons
Core role	Motor output	Executive and social network integration	Homeostatic regulation	Local circuit modulation	Sensory processing
Location	Primary motor cortex, brainstem, anterior horn cells	Frontal and temporal cortex	Hypothalamic nuclei	Distributed throughout CNS	Sensory cortex (visual, auditory, somatosensory)
Cell body size	Very large	Large	Small–medium	Small	Medium
Axon length	Very long	Long-range projections	Short–medium	Very short	Medium–long
Output/Input pattern	Efferent output	Long-range projection neurons	Autonomic and endocrine outputs	Local circuit connectivity	Predominantly afferent input
Firing/reactivity	Tonic or phasic firing	Rapid integrative activity	Tonic regulatory firing	Fast local signaling	Rapid stimulus-evoked firing
Network role	Major output nodes	Hub neurons in cognitive networks	Autonomic and endocrine regulation	Timing and inhibitory control	Input integration in cortical circuits
Metabolic load	Very high	Very high	High	Low–moderate	High
Mitochondrial demand	Very high	High	High	Low	High
Calcium burden	High, limited buffering	High synaptic activity	Regulated calcium signaling	Low	High synaptic activity
Proteostasis demand	High	High (RNA processing)	High (peptide synthesis)	Low	High synaptic turnover
Oxidative stress vulnerability	Very high	High	Moderate–high	Low	Moderate–high
ER stress/UPR	Very high	High	Moderate	Low	Moderate
Metabolic stress vulnerability	Very high	High	Moderate–high	Low	Moderate–high

Table 1 summarizes the findings reported in references [29,30,31,32].

**Table 2:** Multisystem involvement in ALS.

System/Feature	Systemic Symptoms	Subclinical Involvement *	Mitochondrial Involvement	Preclinical Involvement †	Comment
General/Sleep	Yes	No	NA	No	Due to hypothalamic dysfunction
Weight Loss	Yes	No	NA	Yes	Due to hypothalamic dysfunction
Cutaneous	No	Yes	Yes	Yes (in fALS carriers)	—
Hepatic	No	Yes	Yes	Not studied	—
Muscle	No	Yes	Yes	Not studied	—
Hematologic	No	Yes	Yes	Not studied	—
Autonomic/General	No	Yes	NA	Not studied	—
Cardiovascular	No	Yes	NA	Not studied	Possible association with hypertension and stroke
Gastrointestinal	No	Unlikely	NA	Not studied	Constipation is common
Exocrine	No	Yes	NA	Not studied	—
Endocrine/Hormonal	No	Yes	NA	Not studied	More closely linked to CNS disease
Immune/Inflammatory	No	Yes	NA	Not studied	More closely linked to CNS disease
Visual/Retinal	No	Yes	NA	Yes	—
Olfactory	No	Yes	NA	Not studied	—

Footnote: * Subclinical involvement indicates that abnormalities are detectable by detailed clinical examination or specialized testing but are not associated with symptoms recognized or reported by the patient. † Preclinical involvement indicates that abnormalities are detectable, either clinically or subclinically, before the onset of overt clinical symptoms.

**Table 3:** Essential minerals and trace elements.

Essential Major Minerals (These Are Required in Amounts More than 100 mg/day But Are Equally Essential.)	Essential Trace Elements (These Are Required in Amounts Less than About 100 mg/day But Are Equally Essential.)
Calcium Phosphorus Magnesium Sodium Potassium Chloride Sulfur	Iron Zinc Copper Manganese Selenium Iodine Molybdenum Cobalt (through vitamin B12) (Possibly chromium)

**Table 4:** Vitamins and their mitochondrial functions.

Vitamin	Major Mitochondrial Functions
Vitamin B1 (Thiamine)	Cofactor for pyruvate dehydrogenase (PDH), α-ketoglutarate dehydrogenase, branched-chain α-ketoacid dehydrogenase
Vitamin B2 (Riboflavin)	Precursor of FAD/FMN; essential for Complex I, Complex II, ETF, fatty acid β-oxidation
Vitamin B3 (Niacin)	Precursor of NAD^+^/NADH and NADP^+^; central to the TCA cycle, oxidative phosphorylation, and sirtuin signaling
Vitamin B5 (Pantothenic acid)	Component of coenzyme A; essential for acetyl-CoA formation, TCA cycle, and fatty acid metabolism
Vitamin B6 (Pyridoxine)	Amino acid metabolism, transamination, mitochondrial one-carbon metabolism
Vitamin B7 (Biotin)	Cofactor for mitochondrial carboxylases (e.g., pyruvate carboxylase)
Vitamin B9 (Folate)	Mitochondrial folate cycle; nucleotide synthesis and one-carbon metabolism
Vitamin B12 (Cobalamin)	Methylmalonic-CoA mutase in mitochondria; odd-chain fatty acid and amino acid metabolism
Vitamin C	Mitochondrial antioxidant; supports iron homeostasis and scavenges ROS
Vitamin E	Lipid-soluble antioxidant protecting mitochondrial membranes from lipid peroxidation
Vitamin E	Lipid-soluble antioxidant protecting mitochondrial membranes from lipid peroxidation
Vitamin K	Emerging role in electron transport and mitochondrial membrane function

**Table 5:** Summary of all the mitochondrial abnormalities across multiple cell types in sporadic ALS, reflecting contributions from non-cell-autonomous toxicity mechanisms [7].

Cell/Tissue	Bioenergetics/Respiratory Chain	Mitochondrial Dynamics/Transport	mtDNA Alterations	Other Mitochondrial Changes
CNS/Motor Neurons	↓ Oxid. Phosl. 31P-MRS [213]; ↓ COX activity [216]; ↓ synaptic mt ATP synthase [217]; ↓ resp. chain gene expression [215]	TDP-43–mediated impair. of axonal mt transport [222]	↑ mutant mtDNA, ↓ mt content [225]; ↓ methyl. mtDNA reg. region with compensatory ↑ mtDNA copy # [221]	↑ mt density in front./temp. lobes [28]; FUS-mediated disruption of mt–ER (MAM) signaling leading to ↓ ATP product. [223]
Skeletal Muscle	↓ COX activity [243]; ↓ Complex I activity [245]; ↑ Complex II/ Citr. synthase ratio; ↓ lactate dehydrog. activity [247].	—	Multiple mtDNA deletions and ↓ mtDNA content [243,245]	Some studies report conflicting findings in muscle mt studies
Skin Fibroblasts	↑ uncoupled mt resp.; ↑ mt memb. potent.; ↓ OCR/ ECAR ratio [233]; hypermetab. state compensating for ↓ ATP [254,255]	—	—	↑ glycolysis indicating altered cellular bioenergetics
iPSC-Derived Motor Neurons	Impaired Oxid. Phosp.; ↓ ATP [261]; shift from glucose-derived pyruvate to fatty-acid util. [259]	**—**	**—**	—
Peripheral Blood Cells (Platelets/Mononuclear Cells/Lymphocytes)	↓ COX activity despite ↑ mito content [58]; Complex I deficiency with ↓ intracellular ATP and compensatory ↑ ADP [59]	—	—	Instability of mt memb. potential, mt depolarization, and ↑ apoptosis in platelets [56]

Abbreviations: accumul. = accumulation; ADP = adenosine diphosphate; citr. = citrate; ATP = adenosine triphosphate; CNS = central nervous system; COX = cytochrome *c* oxidase; dehydrog. = dehydrogenase; DNA = deoxyribonucleic acid; ECAR = extracellular acidification rate; ER = endoplasmic reticulum; front. = frontal; FUS = fused in sarcoma; impair. = impairment; iPSC = induced pluripotent stem cells; MAM = mitochondria-associated membrane; memb. = membrane; methyl. = methylation; MRS = magnetic resonance spectroscopy; mt = mitochondria; OCR = oxygen consumption rate; Oxid. Phosl. = oxidative phosphorylation; potent. = potential; reg. = regulatory; resp. = respiratory; temp. = temporal; TDP = TAR DNA-binding protein. Complex I = NADH dehydrogenase; Complex II = Succinate dehydrogenase; Complex IV = Cytochrome *c* oxidase, ↑ = increased or elevated; ↓ = decreased or lowered.

**Table 6:** Mitochondrial abnormalities in common familial ALS (fALS).

fALS Gene	% of fALS	Bioenergetics	Dynamics/Transport	Mitophagy/Quality Control	mtDNA/Structural Changes
C9orf72	~30–40% (5–10% sALS)	↑ mt memb. potent; ↑ O_2_ consump; ↑ ATP; ↑ ROS; impaired Complex I [262,263,264]; ↓ metabolic flexibility; impaired utilization of adenosine, fructose, and glycogen in fibroblasts and astrocytes [233]	—	Disrupted ER–mt tethering/MAM signaling [265]	Dysregulated mt transcripts in spinal MNs; ↑ mtDNA SNV burden in astrocytes; mtDNA copy # unchanged [235,266]. Metformin ↓ toxic poly-GR, restoring mt function [267]
SOD1	~15–20% (~1–2% sALS)	↑ mt memb. potent; mt hypermet.; ↓ COX activity [262,263,264,265,266,267,268]	Disrupted mt distrib. & size homeost [269]	—	—
TARDBP (TDP-43)	~3–5%	↑ mt memb. potent; hypermet [262,263] ↑ mt resp, ↑ energy-metab [270] ↓ memb. potent, & resp, COX, OS [271]	Altered mt morph. & ↑ motility; ↓ mt transp in axons [272]	mtDNA release due to mt TDP-43 accumul. [260]	—
FUS	~3–5%	Impair. energy metab. [262]	↓ mt motility; ↓ mt size [272,273]	—	↑ mtDNA damage and mutations [274]
OPTN	~1–3%	—	Impaired microtubule-dependent mt axonal transport [275]	Defective OPTN-mediated mitophagy [276]	—
VCP	~1–2%	Mt hypermet; uncoupl. [277,278]	—	Defective autop–lysos. pathw. [279]	Second. mt dysf. ER stress and TDP-43 path. [240]
TBK1	~1–2%	—	—	Impaired mitop/autop signal [280]	—
ANG	~1%	No clear mt impair. [281]	—	—	—
SQSTM1 (p62)	~1%	—	—	Impaired selective mitoph/autoph [282,283]	—
UBQLN2	<1%	—	—	Impair. proteas degrad & mt turnover [284]	—
SIGMAR1	rare	↓ ATP [285]	—	—	ER stress-associated mt injury (Cristae.)
CHCHD10	rare	↓ mt memb. potent.; hypermet. phenotype [262]	—	—	Struct defects reported in CHCHD10-associated disease

Abbreviations: accumul. = accumulation; autoph–lysos. = autophagy-lysosome; COX = cytochrome *c* oxidase; crist.= cristae; degrad. = degradation; distrib.= disrupted; DNA = deoxynucleic acid; dysf. = dysfunction; ER = endoplasmic reticulum; homeost. = homeostasis; hypermet. = hypermetabolism; impair.= impairment; lysos = lysosome; MAM = mitochondria-associated membrane; mt = mitochondria; mtDNA = mitochondrial DNA; memb. = membrane; metab. = metabolism; mitoph-autoph. = mitophagy-autophagy; morph. = morphology; MNs = motor neurons; OPTN = optoneurin; OS = oxidative stress; potent. = potential; prot. = protease; path. = pathway; resp. = respiration; SNV = single-nucleotide variant; struct. = structure; transp. = transportation; ROS = reactive oxygen species; uncoupl. = uncoupling; ↑ = increased or elevated; ↓ = decreased or lowered. Complex I = NADH dehydrogenase.

**Table 7:** Past therapies attempted for protecting mitochondria.

Name (Chronological Order)	Principal Mitochondrial Mechanism	Mitochondrial Target	Clinical Status/Outcome/Other Comments
Branched-chain amino acids	Metabolic substrate	TCA cycle and bioenergetic	No benefits [311,312,313]
Creatine	ATP buffer	Bioenergetics	No benefits [311,312,313]
Coenzyme Q10	Electron transport chain electron carrier	Electron transport	No benefits [311,312,313]
Acetyl-L-carnitine	Fatty acid transporter (β-oxidation enhancer)	Fatty acid transport	No benefits [311,312,313]
Edaravone	Antioxidant (free radical scavenger)	Oxidative stress	FDA/PMDA approved [302,303]
Rasagiline	Anti-apoptotic MAO-B inhibitor	Mitochondrial membrane	No benefits [311,312,313]
Olesoxime	Mitochondrial permeability transition pore stabilizer	Mitochondrial outer membrane	No benefits [311,312,313]
Dexpramipexole	Bioenergetics	ATP production	No benefits [312,314]
Methycobalamin (ultra-high dose)	Indirect mitochondrial protection (methyl donor)	Methylation/oxidative stress	PMDA approval. It is a supplement in the USA and EU [200,315]
Deferiprone	Iron chelation	Mitochondrial iron	No benefits [183,316]
AMX0035 (sodium phenylbutyrate + taurursodiol)	ER–mitochondria protection	MAM/mitochondrial membrane	Positive phase 2 [317,318] but withdrawn (Docket No. FDA-2025-N-2654) after a negative phase 3 study
CNM-Au8	Catalytic gold nanocrystal (bioenergetic enhancer)	NADH oxidation/ATP synthesis	No benefits [318,319]
EPI-589 (vatiquinone)	Redox cycling antioxidant	Oxidative stress/mitochondrial preservation	No benefits [320]

**Table 8:** Mitochondrial functional categories and potential therapeutic targets.

Categories	Descriptions
I. Bioenergetics and Energy Metabolism	Oxidative phosphorylation (OXPHOS); electron transport chain (Complexes I–V); ATP synthesis; NAD^+^/NADH homeostasis; tricarboxylic acid (TCA) cycle; glycolysis–mitochondrial coupling; mitochondrial pyruvate carrier (MPC); fatty acid transport and β-oxidation; ketone metabolism; amino acid metabolism/anaplerosis; metabolic flexibility
II. Oxidative Stress and Redox Homeostasis	Reactive oxygen species (ROS); antioxidant defense systems (SOD2, glutathione, thioredoxin, peroxiredoxins); iron homeostasis; copper homeostasis; cellular redox signaling
III. Membrane Integrity and Ion Homeostasis	Mitochondrial membrane potential (ΔΨm); cardiolipin remodeling; mitochondrial permeability transition pore (mPTP); calcium homeostasis; cytochrome *c* release; phospholipid metabolism; cholesterol transport
IV. Mitochondrial Dynamics	Mitochondrial fusion; mitochondrial fission; cristae organization; mitochondrial morphology; mitochondrial network remodeling
V. Mitochondrial Genetics	mtDNA replication; mtDNA repair; mtDNA integrity; heteroplasmy; mitochondrial gene expression
VI. Mitochondrial Quality Control	Mitophagy; mitochondrial quality control; mitochondrial biogenesis; mitochondrial protein quality control; mitochondrial unfolded protein response (UPR_mt_); mitochondrial proteostasis; mitochondrial protein import (TOM/TIM complexes); * mitochondrial RNA metabolism; * mitochondrial ribosomal function
VII. Intracellular Mitochondrial Trafficking and Organelle Communication	Axonal mitochondrial transport; dendritic mitochondrial transport; mitochondrial anchoring; endoplasmic reticulum–mitochondria contact sites (MAMs); * mitochondria–lysosome communication; * mitochondria–peroxisome communication
VIII. Cell survival and death pathways	Intrinsic apoptosis; necroptosis; mitochondrial innate immune signaling (MAVS, cGAS–STING); * ferroptosis; * parthanatos; * inflammatory cell death;
IX. Intercellular mitochondrial communication	* Astrocyte–motor neuron metabolic coupling; * oligodendrocyte–axon metabolic support; * microglial mitochondrial activation; * intercellular mitochondrial transfer; neuron–glia signaling
X. Systemic Mitochondrial Metabolism	* Skeletal muscle mitochondria; * liver mitochondria; adipose tissue metabolism; * immune-cell mitochondria; * endocrine regulation of mitochondrial metabolism

Table 8 summarizes the findings reported in references [321,322,323,324,325,326,327]. Established mitochondrial processes are supported by broad experimental evidence and scientific consensus. * Indicates processes for which the experimental evidence is still emerging.

## Data Availability

No new data were created or analyzed in this study. Data sharing is not applicable to this article.
